# Methionine intervention induces PD-L1 expression to enhance the immune checkpoint therapy response in MTAP-deleted osteosarcoma

**DOI:** 10.1016/j.xcrm.2025.101977

**Published:** 2025-02-20

**Authors:** Haoran Mu, Qi Zhang, Dongqing Zuo, Jinzeng Wang, Yining Tao, Zhen Li, Xin He, Huanliang Meng, Hongsheng Wang, Jiakang Shen, Mengxiong Sun, Yafei Jiang, Weisong Zhao, Jing Han, Mengkai Yang, Zhuoying Wang, Yu Lv, Yuqin Yang, Jing Xu, Tao Zhang, Liu Yang, Jun Lin, Feng Tang, Renhong Tang, Haiyan Hu, Zhengdong Cai, Wei Sun, Yingqi Hua

**Affiliations:** 1 Department of Orthopedic Oncology, Shanghai General Hospital, Shanghai Jiao Tong University School of Medicine, Shanghai, China; 2 Shanghai Bone Tumor Institution, Shanghai, China; 3 Department of Biochemistry and Molecular Cell Biology, Shanghai Key Laboratory for Tumor Microenvironment and Inflammation, Shanghai Jiao Tong University School of Medicine, Shanghai, China; 4 National Research Center for Translational Medicine at Shanghai, State Key Laboratory of Medical Genomics, Shanghai Institute of Hematology, Ruijin Hospital, Shanghai Jiao Tong University School of Medicine, Shanghai, China; 5 Department of Pathology, Shanghai General Hospital, Shanghai Jiao Tong University School of Medicine, Shanghai, China; 6 The Drug and Device Phase I Clinical Research Ward/Demonstration Research Ward of Shanghai Sixth People’s Hospital, Shanghai Jiao Tong University School of Medicine, Shanghai, China; 7 State Key Laboratory of Neurology and Oncology Drug Development, Nanjing, China; 8 Jiangsu Simcere Pharmaceutical Co., Ltd., Nanjing, China; 9 Simcere Zaiming Pharmaceutical Co., Ltd., Shanghai, China; 10 Department of Laboratory Animal Center, Shanghai General Hospital, Shanghai Jiao Tong University School of Medicine, Shanghai, China

**Keywords:** osteosarcoma, immunotherapy, MTAP deletion, tumor microenvironment, methionine metabolism, MAT2A, PD-L1, IKZF1

## Abstract

Osteosarcoma (OS), a malignant bone tumor with limited treatment options, exhibits low sensitivity to immune checkpoint therapy (ICT). Through genomics and transcriptomics analyses, we identify a subgroup of OS with methylthioadenosine phosphorylase (MTAP) deletion, which contributes to ICT resistance, leading to a “cold” tumor microenvironment. MTAP-deleted OS relies on methionine metabolism and is sensitive to methionine intervention, achieved through either dietary restriction or inhibition of methionine adenosyltransferase 2a (MAT2A), a key enzyme in methionine metabolism. We further demonstrate that methionine intervention triggers programmed death-ligand 1 (PD-L1) transcription factor IKAROS family zinc finger 1 (IKZF1) and enhances PD-L1 expression in MTAP-deleted OS cells. Methionine intervention also activates the immune-related signaling pathways in MTAP-deleted OS cells and attracts CD8^+^ T cells, thereby enhancing the efficacy of ICT. Combining methionine intervention with ICT provides a significant survival benefit in MTAP-deleted OS murine models, suggesting a rationale for combination regimens in OS ICT.

## Highlights

• Single-cell atlas of MTAP-del osteosarcoma subtype reveals CD8A^+^ T cell deficiency in TME
• Methionine restriction combined with ICT enhances CD8A^+^T cell infiltration
• MAT2A inhibitor improves ICT efficacy through stimulation of PD-L1 expression

## Graphical abstract

**Figure:**
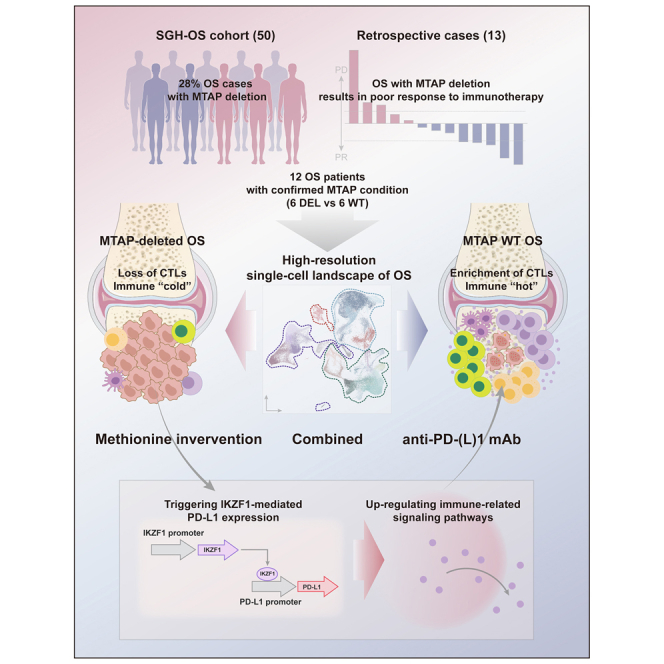

## Teaser

Mu et al. present a high-resolution single-cell atlas of MTAP-deleted osteosarcoma, revealing deficiency in CD8A^+^ T cells. Methionine restriction or MAT2A inhibitor SCR6639 increases CD8A^+^ T cell infiltration in the tumor microenvironment, enhancing the efficacy of immune checkpoint therapy in MTAP-deleted osteosarcoma.

## Introduction

Osteosarcoma (OS) is among the most aggressive bone tumors and is recognized as one of the primary malignant cancers, often afflicting individuals in their first decade of life.^1^^,^^2^ Patients diagnosed with OS typically face a challenging prognosis, and, unfortunately, the available treatment options to enhance their survival are limited. Given the successes in immunotherapy for challenging solid tumors, there’s growing interest in exploring its application for OS treatment. But immunotherapy, especially immune checkpoint therapy (ICT), alone or in combination, failed to achieve the predefined target.^3^^,^^4^ Unlike Ewing sarcoma or liposarcoma, OS exhibits a deficiency in tumor-infiltrating lymphocytes (TILs).^5^ Consequently, elucidating the mechanisms underlying the recruitment and activation of TILs in the OS tumor microenvironment (TME) is imperative. Identifying potential intervention targets is urgently needed to enhance the therapeutic efficacy of OS immunotherapy. In addition to employing programmed death-ligand 1 (PD-L1) expression or tumor mutation burden (TMB) for predicting tumor response to ICT, recent research suggests that immunogenomic characteristics may decrease the abundance of TILs and contribute to primary resistance to ICT.^6^^,^^7^^,^^8^ This introduces a potential therapeutic avenue for enhancing the effectiveness of immunotherapy in the context of OS.

In recent studies, a remarkably high frequency of chromosome 9p21.3 (Chr9p21.3) deletion has been identified in the genomic profile of bone tumors, including OS.^9^^,^^10^^,^^11^^,^^12^^,^^13^ Cyclin-dependent kinase inhibitor 2A (CDKN2A), a crucial tumor suppressor gene located at Chr9p21.3, often experiences loss of function due to genomic deletion in this region. The co-deletion of an adjacent gene, methylthioadenosine phosphorylase (MTAP), is a common occurrence, observed in around 15% of all cancers.^14^^,^^15^ MTAP catalyzes the conversion of S-methyl-5′-thioadenosine (MTA) into adenine and 5-methylthioribose-1-phosphate. This enzymatic activity plays a crucial role in polyamine metabolism and contributes significantly to the salvage pathways of both adenine and methionine. In our previous study, we established a clinical cohort Shanghai General Hospital—Osteosarcoma (SGH-OS) through multi-omics analysis and we identified a frequent genomic Chr9p21.3 deletion in OS^16^ leading to MTAP deletion in OS, consistent with previous study.^17^

Recent research indicates that MTAP-deleted tumor cells exhibit specific vulnerabilities to MAT2A inhibition.^14^^,^^15^^,^^18^^,^^19^ Methionine adenosyltransferase 2a (MAT2A) is a crucial metabolic enzyme involved in methionine metabolism and tumor-related epigenetics.^20^^,^^21^^,^^22^ An explanation of the vulnerability is that the catalytic activity of protein arginine methyltransferase 5 (PRMT5) is sensitive to MAT2A inhibition in MTAP-deleted cells,^14^ though some studies have demonstrated that the accumulated MTA in MTAP-deleted cells is not significantly detected *in vivo*^23^ and the sensitivity of MAT2A or PRMT5 inhibition might be specific in different tumor cells.^15^^,^^20^^,^^24^^,^^25^^,^^26^ However, in MTAP-deleted OS, the role of methionine metabolism and whether intervening it could enhance ICT are still unclear.

Based on the SGH-OS cohort established in our previous multi-omics study,^16^ we focus on enhancing ICT in MTAP-deleted OS in this study, by elucidating its immunogenomic features. We retrospectively followed up with OS patients who underwent ICT and identified their MTAP condition based on next-generation sequencing (NGS) or pathological histology. We analyzed genomic, transcriptomic, and pathological data in our SGH-OS cohort to elucidate the characteristics of MTAP deletion. And we utilized single-cell transcriptome to analyze the reasons for a “cold” immune TME in MTAP-deleted OS. To address this, we demonstrated the effectiveness and mechanisms of methionine intervention in enhancing immunotherapy in MTAP-deleted OS. We further employed a selective MAT2A inhibitor, SCR6639, to enhance the sensitivity of ICT in MTAP-deleted OS.

## Results

### Patients with MTAP deletion exhibit a poor response to immune checkpoint inhibitor therapy

**Figure 1:**
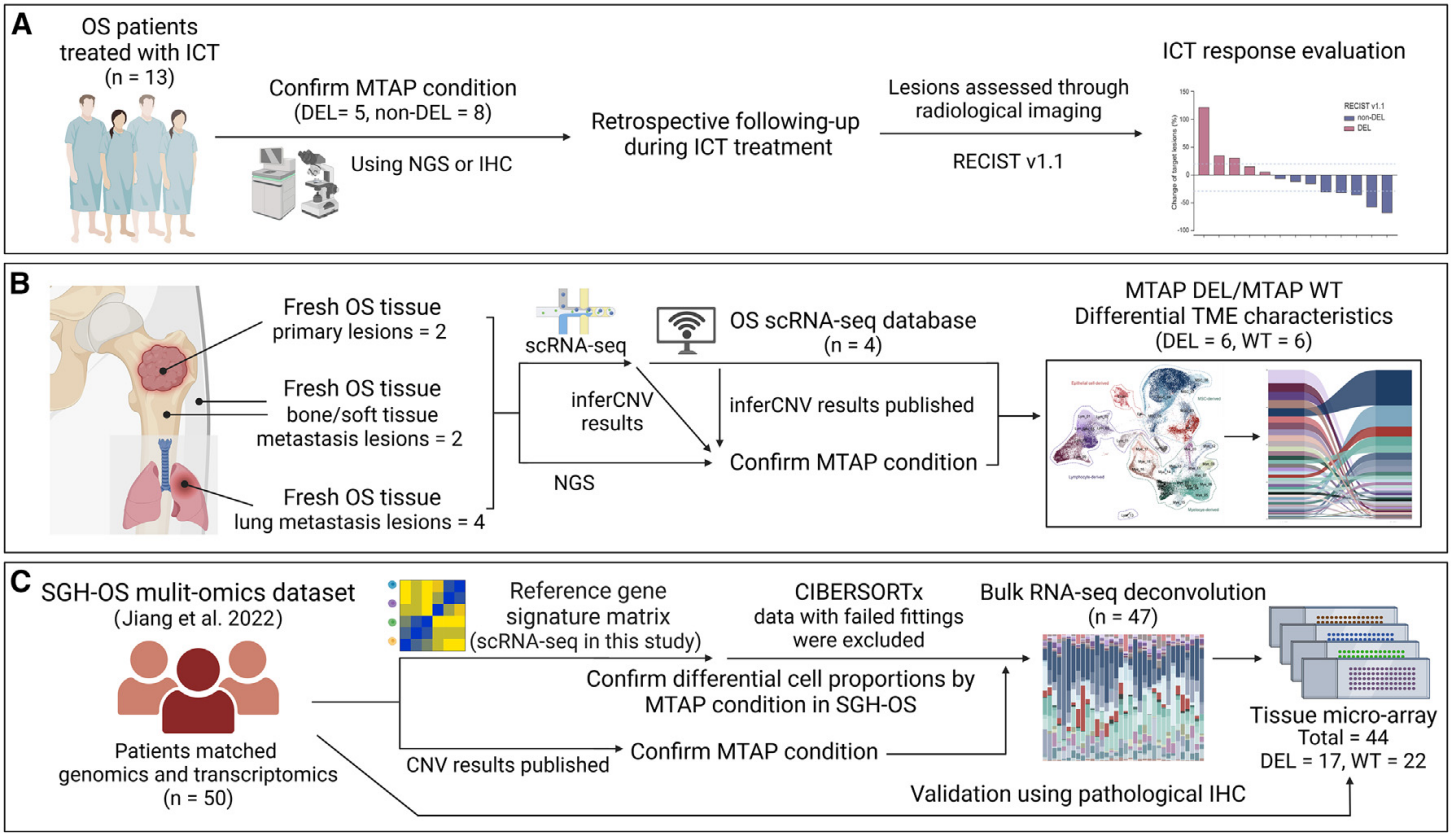
Workflow diagram of an osteosarcoma clinical cohort based on MTAP condition (A) Flowchart of the retrospective immunotherapy follow-up dataset. In brief, 13 osteosarcoma patients who received ICT treatment were categorized based on MTAP status into MTAP deletion (DEL, *n* = 5) and MTAP non-deletion (non-DEL, *n* = 8) groups. Treatment responses were evaluated using imaging data during the ICT course according to RECIST v.1.1 criteria. (B) Flowchart of the single-cell transcriptomic dataset. In brief, fresh single-cell tissues (*n* = 8) and data from an online database (*n* = 4) were obtained. Patients were grouped based on MTAP status, and the tumor microenvironment (TME) was assessed. (C) Flowchart of the matched transcriptomic and genomic dataset. In brief, 50 patients with matched genomic and transcriptomic data from the SGH-OS multi-omics dataset^16^ were categorized according to MTAP status. Deconvolution was performed based on single-cell annotations (successful deconvolution cases, *n* = 47), and validation was conducted using tissue microarray immunohistochemistry (IHC, *n* = 44).

To evaluate the impact of MTAP status on immunotherapy in OS, our study constructed a clinical cohort based on MTAP status, including a retrospective immunotherapy follow-up dataset (Figure 1A), a single-cell transcriptomic dataset (Figure 1B), and a matched transcriptomic and genomic dataset from our previous study (SGH-OS cohort, *n* = 50)^16^ (Figure 1C). These 3 datasets were used to elucidate the effects of MTAP status on OS immunotherapy and the (TME. First, we validated the impact of MTAP status on clinical immunotherapy outcomes in our retrospective immunotherapy follow-up dataset. We included a total of 13 OS patients who received ICT at our center. Based on NGS or immunohistochemistry (IHC) results, the patients were categorized into MTAP deletion (*n* = 5) and MTAP non-deletion (*n* = 8) groups (Table S1).

**Figure 2:**
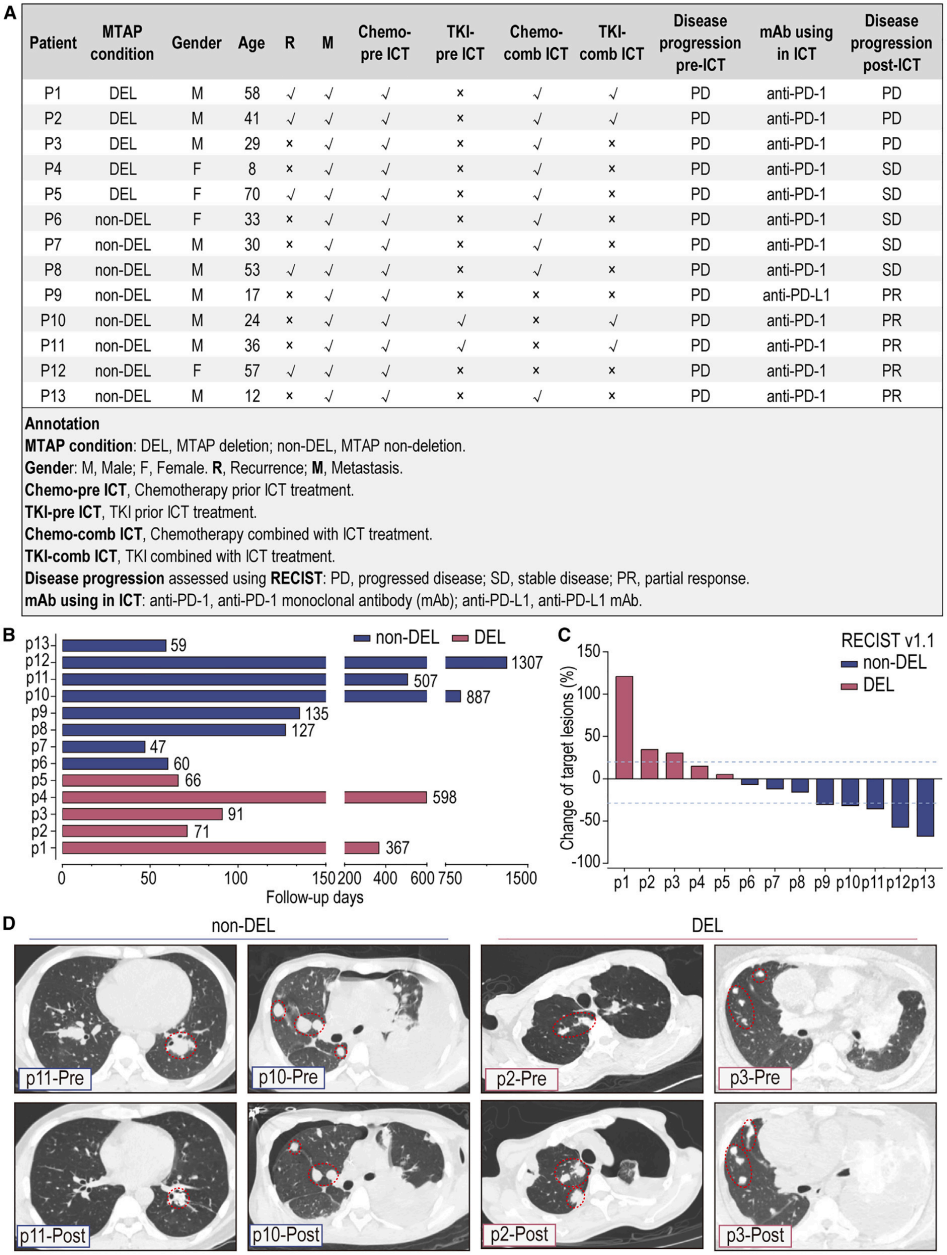
Patients with MTAP deletion exhibit a poor response to immune checkpoint inhibitor therapy (A) The clinical information of the retrospective immunotherapy follow-up cohort. The patients were categorized into MTAP deletion (*n* = 5) and MTAP non-deletion (*n* = 8). Detailed patient information was provided in Table S1. (B) Follow-up days of each patient in this retrospective immunotherapy follow-up cohort. Red represents the DEL group, while blue represents the non-DEL group. (C) The RECIST v.1.1 scores of the 13 patients. Red represents the DEL group, while blue represents the non-DEL group. (D) Imaging characteristics of patients in each group. Pre, before immunotherapy; Post, after immunotherapy at the end of follow-up.

Follow-up assessments included MTAP condition, gender, age, primary site, recurrence (R) or metastasis (M) status, treatments pre-ICT, treatments combined with ICT, disease progression pre- and post- ICT, and follow-up days (Figures 2A and 2B). All patients in the MTAP deletion group (DEL) determined their MTAP status using NGS. This group included 3 males and 2 females, with an average age of 41.2 years (±24.3). Among them, 2 had tumors located in the limbs, 3 in the pelvis, and 1 in another location. All 5 patients had metastases: 2 had lung metastases, 3 had metastases in locations other than the lungs, and 1 of the 5 had multiple metastases. In addition, 3 patients experienced recurrence. At the start of ICT, all patients were undergoing or had experienced postoperative chemotherapy. All 5 patients received anit-PD-1 monoclonal antibody (anti-PD-1 mAb) therapy with chemotherapy, with 2 of them also using tyrosine kinase inhibitors (TKIs). In the MTAP non-deletion group (non-DEL), MTAP condition was determined using NGS for 4 patients and IHC for 4 patients. This group included 6 males and 2 females, with an average age of 32.8 years (±15.9). Among them, 3 had tumors located in the limbs, 4 had tumors located in the pelvis, and 1 in another location. All 8 patients had metastases: 8 had lung metastases, and 1 of the 8 had multiple metastases. Additionally, 2 patients experienced recurrence. At the start of ICT, all patients were undergoing or had experienced postoperative chemotherapy, with 2 of them undergoing TKI treatment. 4 patients received a combination of chemotherapy and ICT, and 2 patients received a combination of TKI and ICT. For monoclonal antibody (mAb) using in ICT, 7 patients received anti-PD-1 mAb therapy and 1 patient received anti-PD-L1 monoclonal antibody (anti-PD-L1 mAb) therapy. There were no significant differences in basic clinical information and treatment methods between the two groups.

The treatment efficacy was assessed through imaging and based on RECIST v.1.1 (Figures 2C and 2D). In the MTAP non-deletion (non-DEL) group, follow-up imaging showed lesion reduction (e.g., p12 and p13), with 3 individuals having stable disease (SD) and 5 having a partial response (PR), including 3 individuals at the boundary between SD and PR. In the MTAP deletion (DEL) group, follow-up imaging showed lesion enlargement (e.g., p2 and p3), with 3 individuals having progressive disease (PD) and 2 having SD. This suggests that, in OS ICT treatment, MTAP status serves as a limiting factor in the response to ICT.

### TME variation of OS by MTAP status

**Figure 3:**
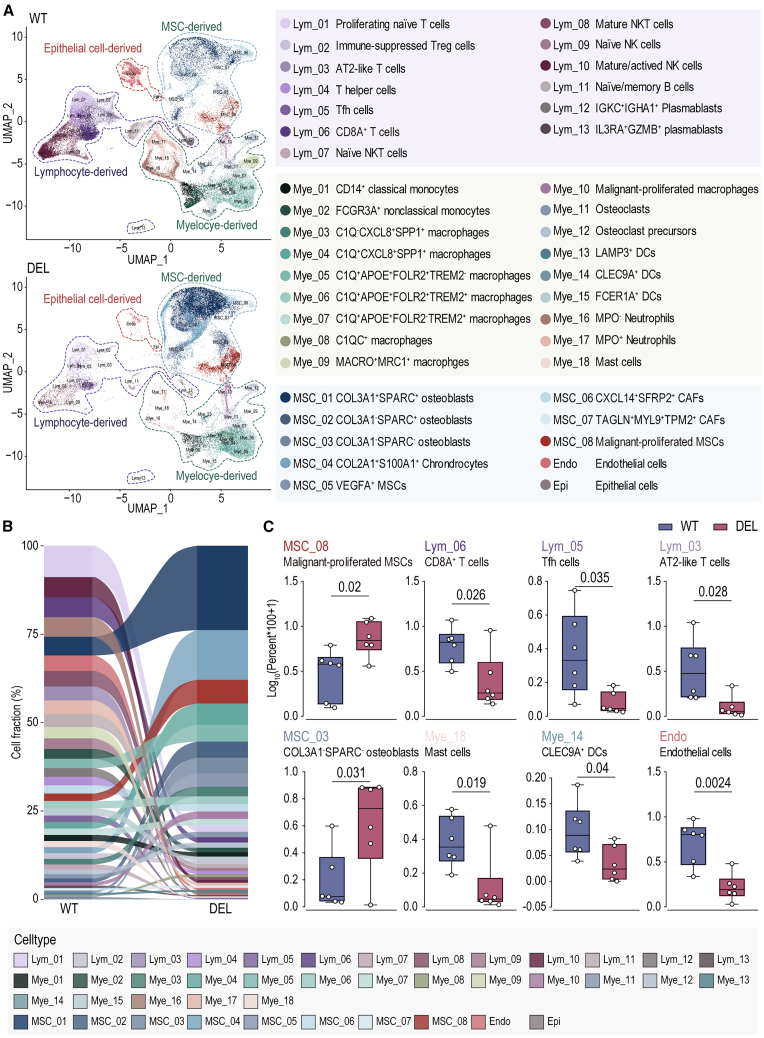
Tumor microenvironment variation of osteosarcoma by MTAP status (A) High-resolution single-cell atlas of the tumor microenvironment in wild-type (WT) and MTAP-deleted (DEL) osteosarcoma. (B and C) Changes in various cell subgroups between WT and DEL were illustrated using a Sankey diagram. Statistical analyses were performed using two-tailed Student’s t tests. 8 cell subgroups exhibited statistically significant differences between WT (*n* = 6) and DEL (*n* = 6).

The effectiveness of ICT is influenced by the TME. To explore whether the efficacy of ICT in OS is related to different TMEs associated with MTAP status, we further utilized the single-cell transcriptomic dataset (*n* = 12) from our clinical cohort to elucidate the OS TME with different MTAP statuses, including 6 metastases, 2 primary tumor lesions, and 4 online data (Table S2; Figure S1A). A high-resolution single-cell OS TME atlas was established, including a total of 100,416 cells. The data were partitioned into 41 cellular subgroups, including 13 lymphoid-lineage subgroups (Lym_01 to Lym_13), 18 myeloid-lineage subgroups (Mye_01 to Mye_18), 8 mesenchymal-origin subgroups (MSC_01 to MSC_08), 1 endothelial subgroup (Endo), and 1 epithelial subgroup (Epi) (Figures 3A, S1B, and S2; Table S3). The atlas was divided into 6 MTAP-deleted (DEL) and 6 wild-type (WT) samples based on clinical NGS, further confirmed using copy-number variation (CNV) calculated by inferCNV (Figure S3). We observed that lymphoid cells displayed minimal MTAP expression, whereas myeloid and mesenchymal stem cell (MSC)-derived cell populations exhibited comparatively higher levels. Importantly, subpopulations expressing MTAP were more prevalent in WT (Figure S4A and S4B). Although overall MTAP expression was low across all cell subpopulations, a comparison between WT and DEL revealed that WT demonstrated detectable MTAP expression, while DEL patients exhibited nearly absent expression (Figure S4C).

We further compared the differences in cellular components between DEL and WT TMEs. The alluvial plot indicates significant differences in cellular composition between DEL TME and WT TME (Figures 3B and S5A; Table S4). 8 cell subpopulations showed statistically significant differences. Malignant-proliferated MSCs (MSC_08) and COL3A1^−^SPARC^−^ osteoblasts (MSC_03) were elevated in the DEL TME, while CD8A^+^ T cells (Lym_06), T follicular helper (Tfh) cells (Lym_05), AT2-like T cells (Lym_03), mast cells (Mye_18), CLEC9A^+^ dendritic cells (DCs, Mye_14), and endothelial cells (Endo) were reduced in the DEL TME (Figure 3C). Cell-cell communication analysis was further performed to determine whether the statistically significant differences in cellular components and their associated molecular signal transduction changes play a role in ICT. Our results revealed that, in the DEL TME, the expression of checkpoint-related receptor-ligand pairs between relevant cells was reduced (Figures S5B and S6). These findings suggest that MTAP status influences the immune microenvironment of OS and weakens molecular signals related to ICT treatment.

### Immunosuppressive TME lacking CD8A^+^ T cells in MTAP-deleted OS

**Figure 4:**
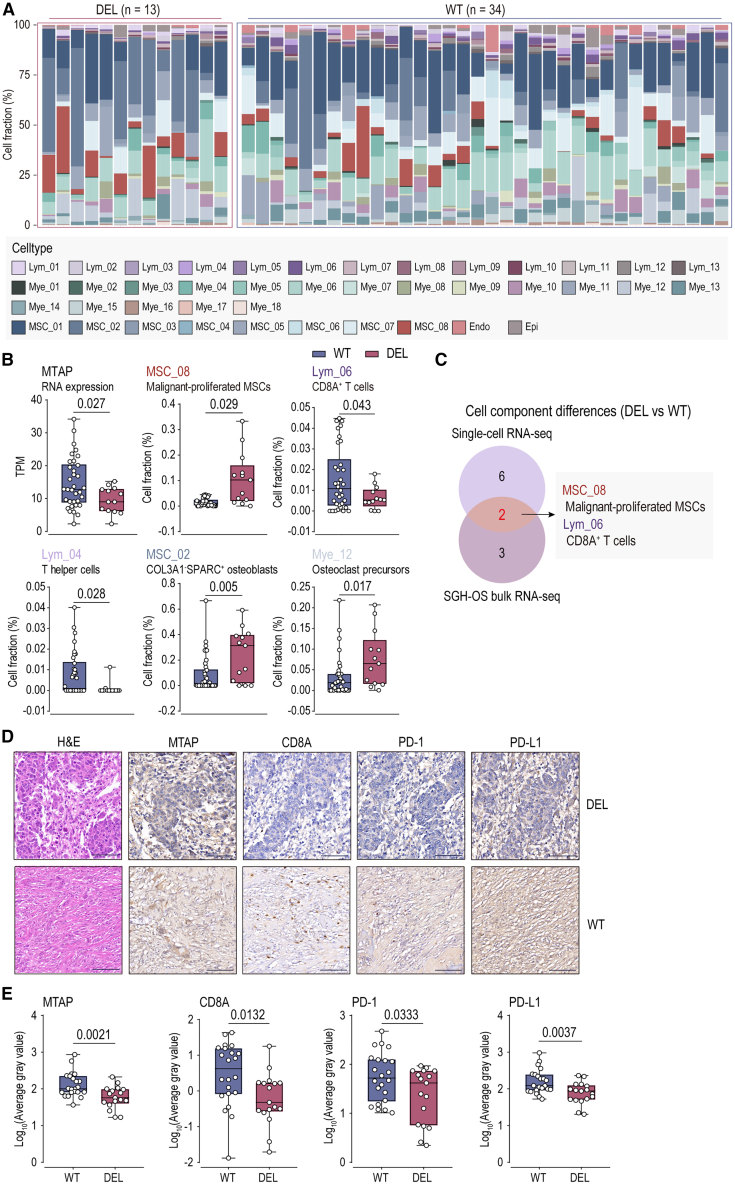
Immunosuppressive tumor microenvironment lacking CD8A^+^ T cells in MTAP-deleted osteosarcoma (A) Deconvolution of matched transcriptomic and genomic datasets of the SGH-OS cohort, comprising 13 MTAP-deleted (DEL) and 34 wild-type (WT) cases, was performed using CIBERSORTx. (B) Statistical analyses were performed using two-tailed Student’s t tests. 5 cell subgroups show statistically significant differences between WT (*n* = 34) and DEL (*n* = 13). (C) Integrated analysis of statistically significant differences between SGH-OS bulk RNA-seq deconvoluted and single-cell RNA-seq in WT and DEL identified Mt (malignant-proliferated cells) and Lym-06 (CD8A^+^ T cells) as differential cell components. (D and E) Pathological characteristics and immunohistochemistry (MTAP, CD8A, PD-1, and PD-L1) of SGH-OS cohort matched tissue microarray, along with quantification. Statistical analyses were performed using two-tailed Student’s t tests. MTAP-deleted (DEL) *n* = 17; wild-type (WT) *n* = 22; Scale bar, 100 μm.

To validate the conclusions drawn from the single-cell transcriptomic dataset, we further utilized the SGH-OS cohort,^16^ which includes matched transcriptomic and genomic data (*n* = 50). In the SGH-OS cohort, genomic features were inferred through CNVs predicted by copy-number arrays. The predicted copy-number loss of MTAP (MTAP-deleted) accounts for 28.00% (*n* = 14), and this CNV further led to the downregulation of MTAP expression (Figures S8A and S8B; Table S5). Based on the single-cell transcriptomic expression matrix, we used deconvolution (CIBERSORTx)^27^^,^^28^^,^^29^ to deconvolve the bulk RNA sequencing (RNA-seq) data. The successfully deconvolved cases included a total of 13 MTAP-deleted (DEL) and 34 WT cases (Figure 4A; Table S6). There remained a statistically significant difference in MTAP RNA expression between the DEL and WT groups. Comparing the differences in cellular components between the two groups, we found 5 cell components with statistically significant differences: malignant-proliferated MSCs (MSC_08), COL3A1^−^SPARC^+^ osteoblasts (MSC_02), and osteoclast precursors (Mye_12) were elevated in the DEL TME, while CD8A^+^ T cells (Lym_06) and T helper cells (Lym_04) were reduced in the DEL TME (Figure 4B).

Combining the analysis of the single-cell transcriptomic dataset and the SGH-OS dataset, we found that only two cell components showed significant differences in both the datasets: malignant-proliferated MSCs (MSC_08) and CD8A^+^ T cells (Lym_06) (Figure 4C). Additionally, in the SGH-OS dataset, the expression of CD8A and related genes was significantly downregulated in the DEL group (Figure S8C). The signaling pathways related to T cell activity were also downregulated in MTAP-deleted OS (Figures S8D and S8E). This suggests that changes in MTAP status primarily affect the distribution of CD8A^+^ T cells and their associated molecular signaling pathways. We further utilized tissue microarray (TMA) paired with the SGH-OS dataset (*n* = 44), including 22 WT and 17 DEL cases based on their genomic characteristics (Figure S9; Table S7). IHC was used to detect the protein expression of ICT-related molecules (CD8A, PD-1, and PD-L1). Compared to WT, the DEL microenvironment was lacking CD8A^+^ T cells and showing downregulated expression of PD-1 and PD-L1 (Figures 4D and 4E). Additionally, the downregulation of MTAP expression was associated with poor prognosis (Figure S8F). This suggests that MTAP status is related to the treatment response of OS, especially in ICT, and MTAP deletion in OS cells affects the activity and distribution of CD8A^+^ T cells, leading to the formation of an immunosuppressive TME.

### Function deficiency by MTAP deletion independently resulting in CD8A^+^ T cell deficiency in OS

**Figure 5:**
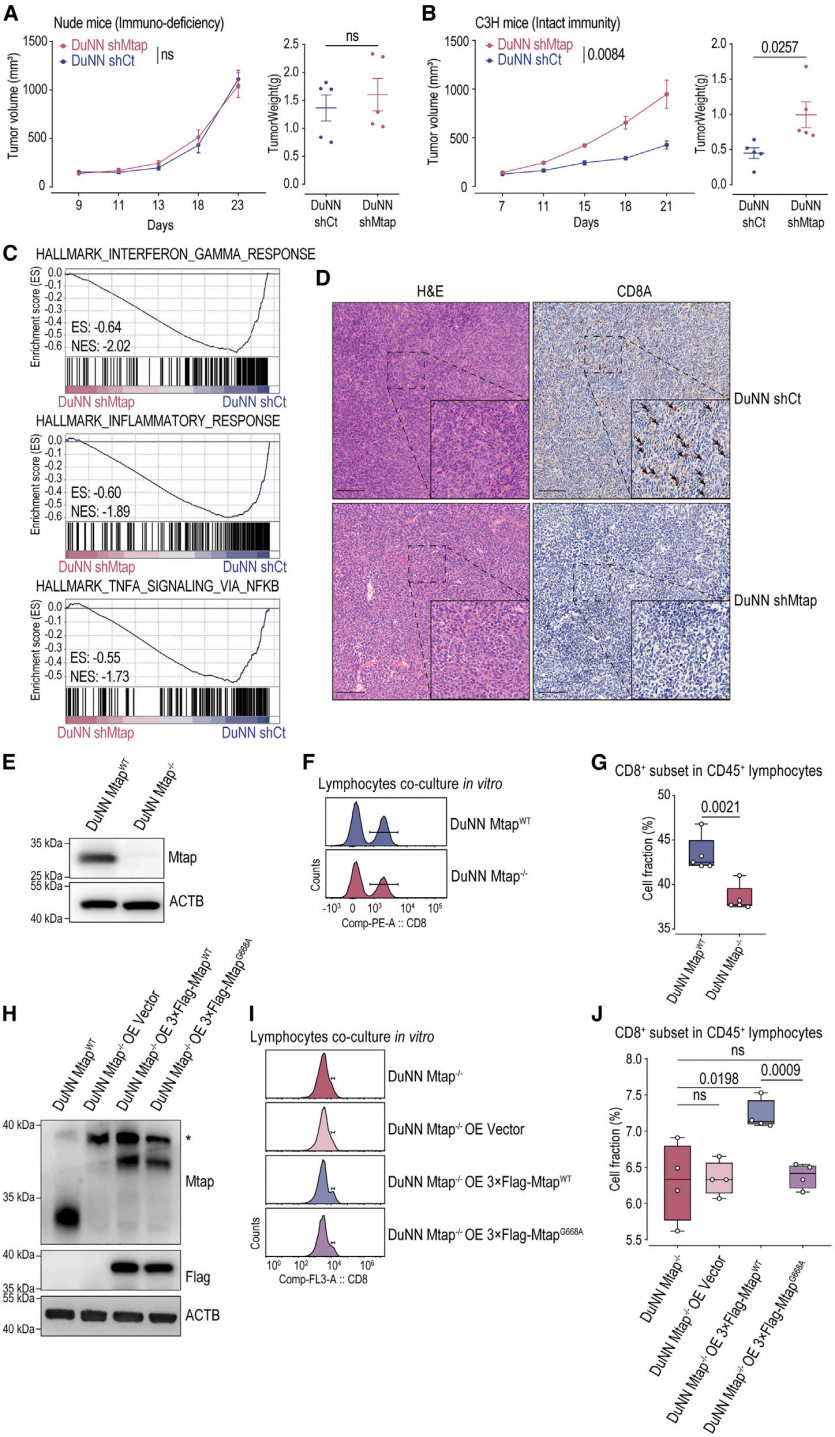
Function deficiency by MTAP deletion independently resulting in CD8A^+^ T cell deficiency in osteosarcoma (A and B) *In vivo* orthotopic tibia models of DuNN shMTAP (*n* = 5) and DuNN shCt (*n* = 5) in nude mice (immunodeficient) and C3H mice (immunocompetent), depicting tumor growth curves (mean ± SEM) and tumor weights at experimental endpoints. Statistical analyses were performed using two-tailed Student’s t tests. ns, not statistically significant. (C) Transcriptomic pathway analysis of tumor tissues from DuNN shMTAP and DuNN shCt in C3H mice, using GSEA analysis on Hallmark gene sets. ES, enrichment score; NES, normalized enrichment score. (D) Pathological characteristics and immunohistochemistry (CD8A) of tumor tissues from DuNN shMTAP and DuNN shCt in C3H mice. Arrows indicate CD8A^+^ T cells. Scale bar, 100 μm. (E) Protein expression of Mtap in DuNN Mtap^WT^ and DuNN Mtap^−/−^ by western blotting. (F and G) The proportion of CD8^+^ T cells among CD3^+^ T cells after co-culture with DuNN Mtap^WT^ (*n* = 5) or DuNN Mtap^−/−^ (*n* = 5) by flow cytometry and quantification. Statistical analyses were performed using two-tailed Student’s t tests. (H) Protein expression of Mtap in DuNN Mtap^WT^, DuNN Mtap^−/−^ OE vector, DuNN Mtap^−/−^ OE 3×FLAG-Mtap^WT^, and DuNN Mtap^−/−^ OE 3×FLAG-Mtap^G668A^ by western blotting. (I and J) The proportion of CD8^+^ T cells among CD45^+^ T cells after co-culture with DuNN Mtap^−/−^ (*n* = 4), DuNN Mtap^−/−^ OE vector (*n* = 4), DuNN Mtap^−/−^ OE 3×FLAG-Mtap^WT^ (*n* = 4), and DuNN Mtap^−/−^ OE 3×FLAG-Mtap^G668A^ (*n* = 4) by flow cytometry and quantification. Statistical analyses were performed using two-tailed Student’s t tests.

To exclude the impact of other genomic features and signaling pathway alterations on the OS TME, we first constructed an MTAP function deficiency mouse OS cell model (DuNN shMtap) and its orthotopic model in the tibia (Figure S10A). Under *in vitro* culture conditions, there was no difference in growth between DuNN shMTAP and its control (DuNN shCt) (Figure S10B). Orthotopic models of DuNN shMtap and DuNN shCt were created in immune-deficiency nude mice and intact-immunity mice (C3H mice). While there was no significant difference in tumor growth in nude mice, a significant distinction emerged in C3H mice between DuNN shMtap and DuNN shCt (Figures 5A, 5B, and S10C). In intact-immunity C3H mice, immune-related signaling pathways were downregulated in DuNN shMtap tumor tissues. This included pathways such as “interferon gamma response,” “inflammatory response,” and “TNFA signaling via NFKB” in gene set enrichment analysis (GSEA) Hallmark gene sets (Figures 5C, S10D, and S10E). Concurrently, tumor proliferation-related signaling pathways, such as “E2F targets” in GSEA Hallmark gene sets, were upregulated, corresponding to accelerated tumor growth (Figure S10F). This suggests an immunosuppressive TME in DuNN shMtap tissues. Further, IHC demonstrated a lack of CD8A^+^ T cells in DuNN shMtap (Figures 5D and S10G), consistent with the TME features observed in clinical OS TMA.

To verify that the function of MTAP independently leads to CD8A^+^ T cell deficiency in OS tissue, we further constructed an Mtap genomic deletion mouse OS cell model (DuNN Mtap^−/−^) and its control (DuNN Mtap^WT^). Additionally, we rescued different functional MTAP proteins into the DuNN Mtap−/− cells, creating cell models with normal Mtap protein (DuNN Mtap^−/−^ OE 3×FLAG-Mtap^WT^) and inactive Mtap protein (DuNN Mtap^−/−^ OE 3×FLAG-Mtap^G668A^) (Figures S11A and S11B), and co-cultured these different cell models with T cells *in vitro* (Figure S11C). In DuNN Mtap^−/−^, Mtap function was completely lost (Figure 5E), and flow cytometry revealed a decrease in the proportion of CD8A^+^ T cells in the co-cultured T cells (Figures 5F and 5G). After functional rescue, different functional Mtap proteins were detected again (Figure 5H). Upon rescue of the normal Mtap^WT^, the proportion of CD8A^+^ T cells in the co-cultured T cells increased. However, upon rescue of the inactive Mtap^G668A^, the proportion of CD8A^+^ T cells in the co-cultured T cells remained decreased (Figures 5I and 5J). These results indicate that MTAP function deficiency in MTAP-deleted OS can independently lead to CD8A^+^ T cell deficiency in TME.

### Reversing CD8A^+^ T cell deficiency by methionine metabolism intervention enhancing immunotherapy response in MTAP-deleted OS

**Figure 6:**
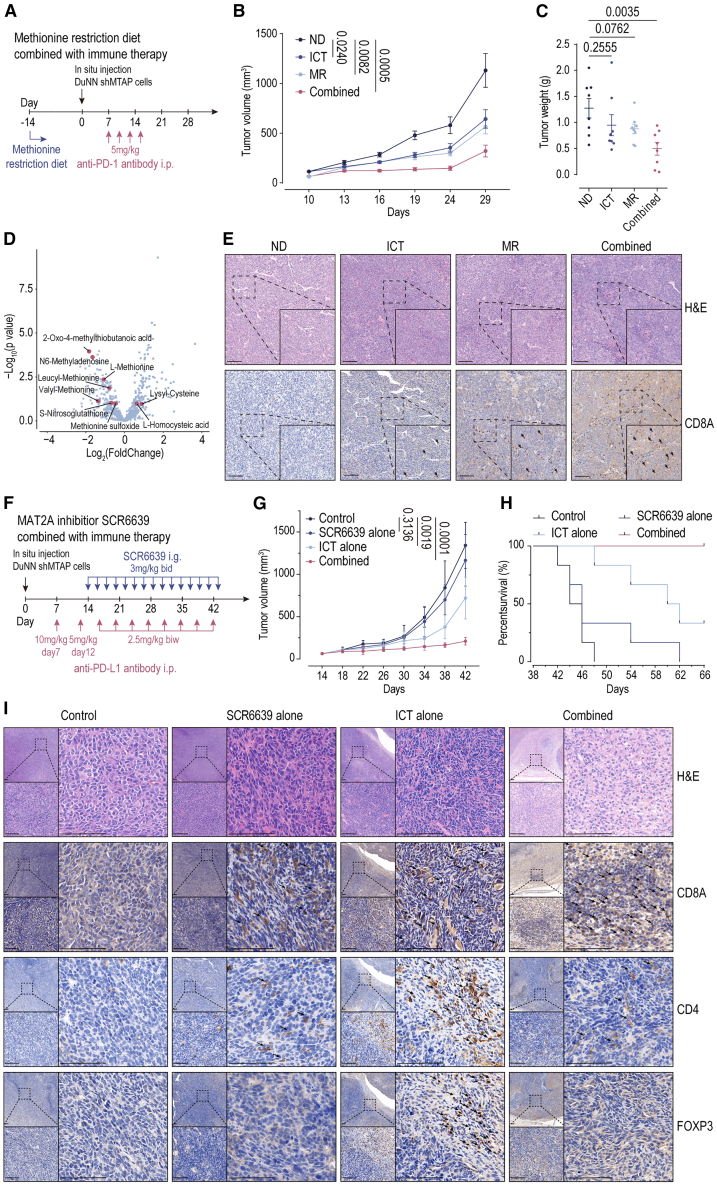
Reversing CD8A^+^ T cell deficiency by methionine metabolism intervention enhancing immunotherapy response in MTAP-deleted osteosarcoma (A) Flowchart depicting methionine restriction diet combined with immune checkpoint therapy. (B and C) *In vivo* orthotopic tibia models of DuNN shMtap in C3H mice, depicting tumor growth curves (mean ± SD) and tumor weights at experimental endpoints, treated by methionine restriction diet combined with immune checkpoint therapy. ND *n* = 8; ICT *n* = 8; MR *n* = 8; Combined *n* = 8. Statistical analyses were performed using two-tailed Student’s t tests. (D) Metabolomics of the intestines in DuNN shMtap tumor-bearing C3H mice revealed downregulation of methionine-related metabolites following dietary methionine restriction. (E) Pathological characteristics and immunohistochemistry (CD8A) of tumor tissues from DuNN shMtap in C3H mice, treated by methionine restriction diet combined with immune checkpoint therapy. Arrows indicate T cells. Scale bar, 100 μm (F) Flowchart depicting MAT2A inhibition combined with immune checkpoint therapy. (G and H) *In vivo* orthotopic tibia models of DuNN shMtap in C3H mice, depicting tumor growth curves (mean ± SD) and survival curve at experimental endpoints, treated by MAT2A inhibition combined with immune checkpoint therapy. ND *n* = 6; ICT *n* = 6; MR *n* = 6; Combined *n* = 6. Statistical analyses were performed using two-tailed Student’s t tests. (I) Pathological characteristics and immunohistochemistry (CD8A, CD4, and FOXP3) of tumor tissues from DuNN shMtap in C3H mice, treated by MAT2A inhibition combined with immune checkpoint therapy. Arrows indicate T cells. Scale bar, 100 μm.

Despite the survival advantage that MTAP deletion confers to the OS TME through T cell deficiency, we identified a related metabolic vulnerability in DuNN shMtap tissues—activation of methionine metabolism (Figure S10H), a pathway previously reported to be synthetically lethal with MTAP deletion.^14^^,^^15^^,^^19^ This indicates that OS cells, while losing MTAP function, require methionine to maintain their immune evasion advantage. Based on this hypothesis, we first investigated whether methionine metabolism intervention through dietary methionine restriction could enhance the sensitivity of MTAP-deleted OS to ICT. We established the DuNN shMtap tibia *in situ* model and administered a combination of a methionine-restricted diet and ICT, using anti-PD-1 antibody, to four groups: Normal diet without ICT (ND), normal diet with anti-PD-1 antibody (ICT), methionine-restricted diet without anti-PD-1 antibody (MR), and combined group with both methionine-restricted diet and anti-PD-1 antibody (Combined) (Figure 6A). Combined exhibits significantly reduced tumor growth, and the endpoint tumor weight is significantly lower than that in ND (Figures 6B, 6C, and S13A). Methionine-restricted diet significantly downregulated methionine-related metabolites in gut metabolomics (Figure 6D) and upregulated CD8A^+^ T cell distribution in the OS TME (Figures 6E and S13B), and these indicate that methionine intervention through methionine-restricted diet can enhance ICT in MTAP-deleted OS.

Considering that OS primarily affects adolescents, widespread methionine restriction might lead to toxic effects such as developmental abnormalities in adolescents. We further considered the inhibition of the key enzyme in methionine metabolism, MAT2A, by a selective inhibitor SCR6639 (Figure S12). We also established the DuNN shMtap tibia *in situ* model and administered a combination of SCR6639 and ICT, using anti-PD-L1 antibody, to four groups: not used SCR6639 or ICT (Control), used SCR6639 alone (SCR6639 alone), used anti-PD-L1 antibody alone (ICT alone), and used SCR6639 in combination with anti-PD-L1 antibody (Combined) (Figure 6F). Combined exhibits significantly reduced tumor growth and significantly extends the survival time of mice (Figures 6G, 6H, and S13C). We assessed the molecular biological changes in DuNN shMtap following ICT enhancement through MAT2A inhibition using transcriptomic analysis. Immune-related signaling pathways were activated by MAT2A inhibition (Figure S13D), but signaling pathways associated with tumor progression, such as cell cycle, DNA repair, R loop disassembly, methylation, methionine biosynthetic process, and histone methylation, were downregulated in response to MAT2A inhibition (Figure S13E). Additionally, a significant overall upregulation in T cells, NK cells, B cells, and myeloid cells (monocytes-macrophages, DC cells), including CD8A^+^ T cells and cytotoxic T lymphocytes, was detected in the combined treatment group with ICT and MAT2A inhibition by MCP counter (Figure S13F). Further, we observed an increase in the proportion of CD8A^+^ T cells in the combined group and a reduction in the proportion of CD4^+^ T cells and FOXP3^+^ T cells (Figures 6I and S13G). These findings suggest that targeting MAT2A for methionine intervention could reverse the CD8A^+^ T cell deficiency in the immune-suppressive microenvironment of MTAP-deleted OS, thereby enhancing its sensitivity to ICT.

### MAT2A inhibition triggering IKZF1-mediated PD-L1 expression in MTAP-deleted OS

**Figure 7:**
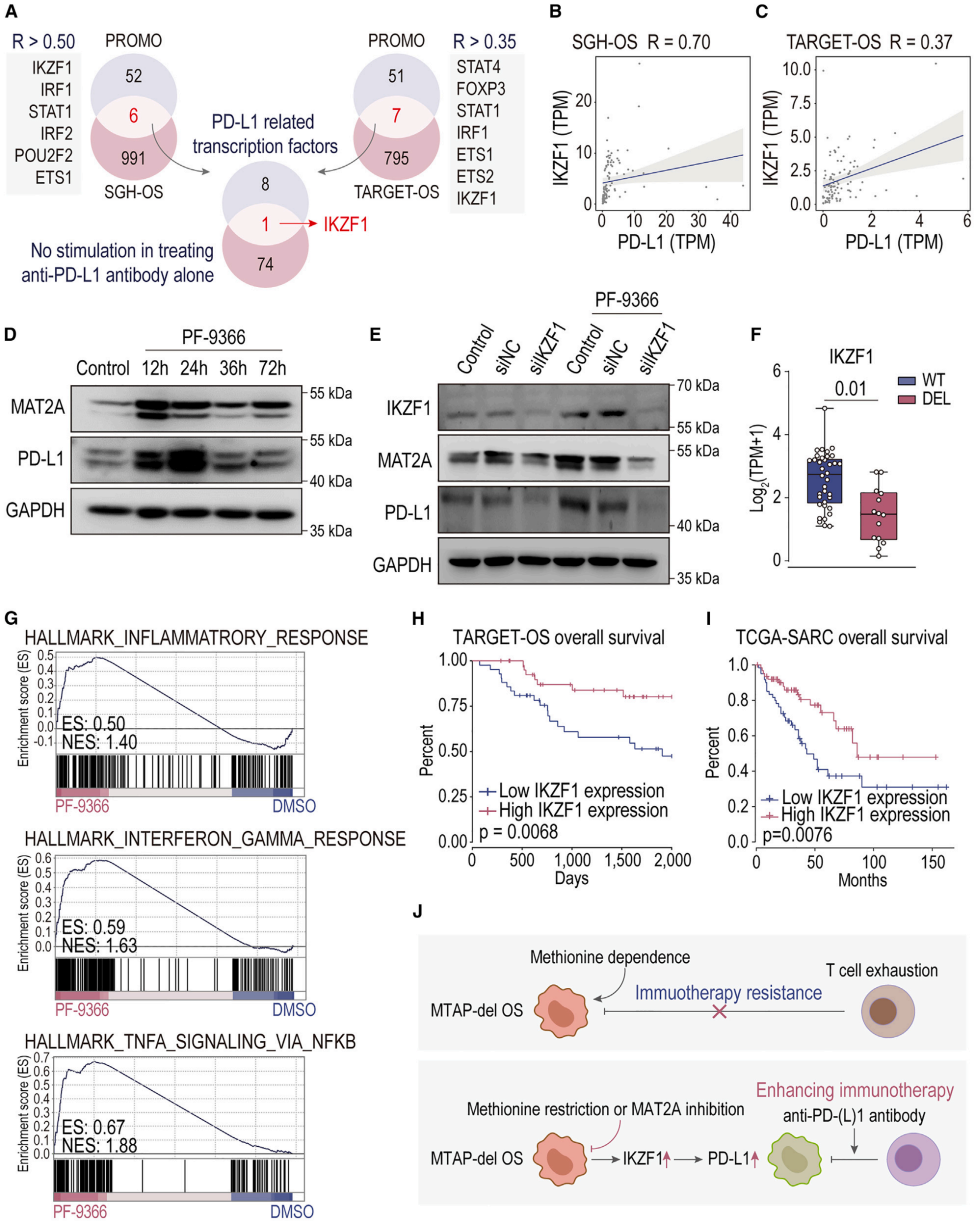
MAT2A inhibition triggering IKZF1-mediated PD-L1 expression in MTAP-deleted osteosarcoma (A, B, and C) Integrated analysis of transcription factors associated with PD-L1 expression in SGH-OS and TARGET-OS and genes upregulated by treating with MAT2A inhibitor rather than immune checkpoint therapy. Spearman correlation tests were performed, and data with *p* values < 0.05 were considered statistically significant. (D and E) Protein expression of MAT2A and PD-L1 in MTAP-deleted OS cells (143B) treated with MAT2A inhibitor PF-9366, and protein expression of IKZF1, MAT2A, and PD-L1 in 143B cells co-treated with siIKZF1, assessed by western blotting. (F) IKZF1 RNA expression in wild-type (WT) (*n* = 36) and MTAP-deleted (DEL) (*n* = 14) cases in SGH-OS cohort. Statistical analyses were performed using two-tailed Student’s t tests. (G) Transcriptomic pathway analysis of cells by treating with PF-9366, using GSEA analysis (*p* value < 0.05) on Hallmark gene sets. ES refers to enrichment score, and NES refers to normalized enrichment score. (H and I) Survival curve of TARGET-OS and TCGA-SARC related with IKZF1 expression. Statistical significance was calculated using the log rank test. (J) Summary schematic illustrating the mechanism of methionine intervention enhancing immune checkpoint therapy in MTAP-deleted osteosarcoma (MTAP-del OS).

We further investigated the potential mechanisms by which MAT2A inhibition constrains immune evasion of MTAP-deleted OS cells. Initially, we analyzed genes associated with PD-L1 expression, a crucial tumor immune checkpoint, within the SGH-OS and TARGET-OS datasets. This analysis, integrated with predictions of PD-L1 transcription factors predicted by PROMO and genes activated in DuNN shMtap tissue transcriptomic following MAT2A inhibition (Figure S14), revealed the functional activation of the PD-L1 transcription factor Ikaros family zinc finger 1 (IKZF1) (Figures 7A, 7B, and 7C). *In vitro* treatment of the MTAP-deleted OS cell line 143B with the MAT2A inhibitor PF-9366 resulted in an upregulation of PD-L1 protein expression (Figure 7D). However, interfering with IKZF1 function using small interfering RNA (siIKZF1) prevented MAT2A inhibition from activating PD-L1 expression (Figure 7E). The *in vivo* IHC staining proved that MAT2A inhibition or methionine diet restriction enhanced IKZF1 expression in tumor tissue (Figure S15). Additionally, analysis of the SGH-OS dataset showed a significant downregulation of IKZF1 expression in MTAP-deleted OS (DEL) compared to WT OS (WT) (Figure 7F). Inhibition of MAT2A activity in 143B cells using PF-9366 led to an upregulation of immune-related signaling pathways (Figure 7G), including the “interferon gamma response,” “inflammatory response,” and “TNFA signaling via NFKB” within GSEA Hallmark gene sets.

Moreover, low IKZF1 expression was identified as a prognostic factor associated with poor outcomes in OS patients (TARGET-OS) and in sarcoma patients (TCGA-SARC) (Figures 7H and 7I). These findings suggest that methionine restriction, through either dietary limitation or MAT2A inhibition, involves IKZF1 in the activation of PD-L1 within MTAP-deleted OS cells. This mechanism restricts tumor cell immune evasion, with the activation of immune-related signaling pathways reversing the immunosuppressive TME and enhancing the sensitivity of MTAP-deleted OS cells to ICT (Figure 7J).

## Discussion

Clinically, OS patients resistant to first-line chemotherapy often experience a low life expectancy, underscoring the need for alternative therapies. Immunotherapy, particularly ICT using PD-(L)1 mAb, has been explored in bone and soft tissue sarcomas, but its efficacy in OS remains limited. This limitation is partly due to the rarity of OS, resulting in small patient cohorts, and partly due to its complex genetic background. Our previous study has highlighted the intricate genomic features of OS through a combined-genomic-and-transcriptomic multi-omics molecular subtype.^16^ Based on our previous study, this study emphasizes the significance of the 9p21.3 chromosomal locus, specifically MTAP deletion, in influencing ICT efficacy, by detailing the high-resolution TME characteristics and underlying molecular mechanisms. MTAP deletion leads to an immunosuppressive TME, with some reports indicating that MTAP knockout (KO) results in the upregulation of PD-L1 in tumor cells,^8^ while others have found significantly reduced PD-L1 staining in tumors with 9p21.3 loss (without distinguishing between CDKN2A and MTAP).^7^ Our findings support the latter, showing decreased PD-L1 expression in MTAP-deleted OS tissues. However, regardless of these differences, the overall conclusion remains consistent: MTAP deletion, and more broadly 9p21.3 loss, leads to reduced T cell infiltration, particularly CD8^+^ T cells, and diminished ICT efficacy.

Methionine metabolism has been recognized as vulnerability in MTAP-deleted tumors.^14^^,^^18^ Targeting methionine metabolism has been reported to enhance the sensitivity of tumors to chemotherapy.^15^ Other vulnerabilities in MTAP-deleted tumors, such as PRMT5,^30^ have been linked to immunotherapy efficacy. MTAP deletion in tumor cells leads to the accumulation of MTA in the microenvironment, which negatively impacts PRMT5 activity in T cells, as elevated MTA levels suppress its function. By using active MTAP enzyme-loaded liposomes to reduce high MTA concentrations in the microenvironment, T cell activity can be restored and enhanced.^31^ In this study, we also observed that PRMT5 levels were higher in certain cell subpopulations within the TME of MTAP-deleted (MTAP-del) OS compared to MTAP WT OS, primarily localized to several mesenchymal-derived cell subpopulations (Figures S7A and S7B). Additionally, in the SGH-OS cohort, patients with MTAP deletions exhibited higher RNA expression of PRMT5 compared to WT patients (Figure S8B). However, in our previous experiments, we observed that the sensitivity to PRMT5 inhibitors in MTAP-deleted cells is not consistent. Our study found that MTAP-deleted OS organoid models are sensitive to PRMT5 inhibition and that PRMT5 inhibitors can enhance the response of OS to immunotherapy.^32^ Yet, we also discovered significant variability in the efficacy of the PRMT5 inhibitor EPZ015666 between two sublines (HOS/MNNG and 143B) derived from the same parental cell line (HOS),^26^ indicating that unknown mechanisms underlying the relationship between MTAP deletion and PRMT5 inhibition remain to be explored.

T cell functionality is not solely dependent on PRMT5-mediated symmetric dimethylation of arginine; the balance between S-adenosylmethionine (SAM) and MTA,^14^ which regulates PRMT5 activity, also plays a crucial role in maintaining T cell activity and differentiation. Exogenous methionine restriction reduces SAM levels, leading to a decrease in histone H3K4me3, which alters T cell growth and activity; this process is critical in shaping T helper cells, particularly Th17 cells.^33^ In T cells, both SAM and MTA levels impact methylation status, with elevated concentrations of either leading to T cell deficiency, and restricting MAT2A in T cells can enhance their activity within the hepatocellular carcinoma microenvironment, thereby inhibiting tumor growth.^34^ The methylation status within T cells is also linked to the function of methionine transporters on the cell surface, with antigen-activated T cells regulating the methylation process by maintaining methionine transport.^35^

Methionine is also critical for tumor cells, which scavenge methionine from the microenvironment to meet the demands of rapid proliferation, a phenomenon known as the Hoffman effect.^36^ Limiting methionine intake can inhibit tumor growth.^37^ In the pathogenesis of mixed-lineage leukemia, inhibition of MAT2A reduces SAM levels, which in turn decreases the activity of disruptor of telomeric silencing 1-like (DOT1L) and PRMT5, demonstrating anti-leukemic effects.^20^ These studies suggest that methionine intervention to enhance immunotherapy could be a viable approach in MTAP-deleted tumors, particularly in OS. Targeting methionine metabolism has been also reported to enhance the efficacy of immunotherapy in tumors without MTAP deletion.^38^ Methionine is essential for adolescent development, and excessive restriction can cause severe side effects, while OS predominantly occurs in adolescents. This necessitates consideration of additional factors when treating specific tumor types, like OS, which often exhibits a high stromal density due to complex MSC subpopulations, leading to lower drug infiltration. To address these issues, our study employs a more targeted approach using a selective MAT2A inhibitor instead of dietary methionine restriction. While our experience suggests that this approach is safe (Figure S16), given the anorexia often experienced during chemotherapy, this dietary strategy still requires refinement.

Additionally, we discovered a distinct regulatory mechanism for ICT enhancement in OS compared to other tumors. Even with methionine restriction, ICT enhancement occurs through different pathways depending on the tumor type. In colorectal cancer, tumors absorb dietary methionine to stabilize PD-L1 mRNA methylation, resulting in high expression of PD-L1 and V-domain Ig suppressor of T cell activation (VISTA) in tumor cells, which suppresses the interferon (IFN)-γ signaling pathway in the microenvironment. Methionine restriction enhances the response to ICT by inhibiting YTHDF1-mediated m6A methylation of PD-L1 and VISTA mRNAs, leading to downregulation of PD-L1 expression and activation of the IFN-γ signaling pathway, thereby increasing ICT sensitivity.^38^ However, other studies have shown that, in colorectal cancer, methionine dietary restriction can simultaneously activate Type-I interferons, such as IFN-α and IFN-β, to enhance major histocompatibility complex class I expression, and Type-II interferon, such as IFN-γ, to increase PD-L1 expression in tumor cells.^39^ The use of recombinant methioninase for localized methionine restriction in tumors has also been shown to upregulate PD-L1 expression in tumor tissues, with its safety profile thoroughly validated.^40^^,^^41^^,^^42^ Methionine metabolism restriction or inhibition of the key enzyme MAT2A has differential effects on PD-L1 expression across various tumor cells. Our study found that methionine restriction upregulates PD-L1 expression in MTAP-del OS. We believe that the enhanced ICT efficacy resulting from dietary restriction or MAT2A inhibition is primarily due to the activation of the IFN-γ signaling pathway, a conclusion supported by our findings. IFN-γ is believed to enhance T cell infiltration within the TME^43^ and activate anti-tumor immune responses,^44^ but its function is also multifaceted, with potential roles in promoting tumor progression.^45^ It is also important to consider the duration of methionine restriction, as intermittent but not continuous dietary methionine deprivation can accelerate ferroptosis by promoting the transcription of ChaC Glutathione Specific Gamma-Glutamylcyclotransferase 1 (CHAC1) and enhance the efficacy of ICT through activation of the IFN-γ signaling pathway, whereas prolonged restriction may reverse this effect.^46^ Although our study did not observe a reduction in ICT efficacy following prolonged restriction, this factor should be carefully considered in future research.

In addition, in OS, we unexpectedly identified that IKZF1, a factor primarily associated with hematologic malignancies,^47^ is linked to the upregulation of PD-L1 expression in OS cells. Through comprehensive analysis correlation validation in two cohorts and mouse tissue transcriptomics, we confirmed its reliability. This IKZF1-mediated upregulation of PD-L1 offers a preliminary explanation for how methionine intervention enhances the efficacy of ICT in MTAP-deleted OS. IKZF1 is a transcription factor typically regarded as a regulator of lymphocyte and myeloid cell development; however, previous studies have reported its association with prognosis in OS patients,^48^ which aligns with our findings. Our research indicates that IKZF1 expression is downregulated in MTAP-deleted OS patients. Notably, bulk RNA-seq from animal experiments combining MAT2A inhibitors and ICT revealed an upregulation of IKZF1 RNA in the combination group, a response not observed with ICT alone. Additionally, *in vitro* experiments demonstrated that MAT2A inhibition can elevate IKZF1 expression in MTAP-deleted OS cells. Furthermore, the downregulation of IKZF1 expression was found to suppress the PD-L1 upregulation induced by MAT2A inhibition, providing initial evidence that the PD-L1 upregulation response to MAT2A inhibition in MTAP-deleted OS cells is related to IKZF1. However, the specific role of IKZF1 in OS remains inadequately explored in this study and warrants further investigation in future research.

### Limitations of the study

Although this study has revealed important characteristics of the MTAP-deleted OS microenvironment, there are still some limitations that need to be addressed. Specifically, MTAP status in this study was determined using bulk NGS genomic sequencing or IHC on whole paraffin sections. While these methods provide a general overview, they do not account for the heterogeneity within specific cell populations. Our CNV analysis of various cell subpopulations showed that not all malignant tumor cell subpopulations exhibited MTAP deletion, highlighting the complexity of the TME. Therefore, the role and potential impact of MTAP WT cells within this microenvironment are not yet fully understood. Further studies focusing on the interplay between WT and MTAP-deleted cells could provide more insights into the tumor’s biology. Moreover, our findings are based on preclinical models, and we have not yet directly validated their clinical applicability. To confirm the practical value of these results, additional clinical trials are necessary. These trials would help determine if the proposed therapeutic strategies can be effectively translated into clinical settings for patients with MTAP-deleted OS.

In addition, we have identified and validated that IKZF1 upregulates PD-L1 expression in MTAP-deleted OS cells in response to methionine restriction or MAT2A inhibition. However, the precise molecular mechanism by which IKZF1 activates PD-L1 expression remains to be elucidated. This pathway requires further exploration through more refined and specific experimental models, which could include targeted genetic manipulation or protein interaction studies. Since MAT2A plays a critical role by converting methionine to the intracellular methyl donor SAM, it raises the possibility that epigenetic regulation or post-translational modifications might be involved in the process of IKZF1-mediated PD-L1 activation. Understanding whether these modifications contribute to the observed effects will be crucial for developing more effective therapeutic interventions. Therefore, future studies should focus on delineating these mechanisms through detailed biochemical and molecular approaches, which may open up avenues for targeting IKZF1 and PD-L1 in MTAP-deleted tumors.

## Resource availability

### Lead contact

Further information and requests for resources and reagents should be directed to and will be fulfilled by the lead contact, Yingqi Hua (hua_yingqi@163.com).

### Materials availability

This study did not generate new unique reagents.

### Data and code availability

• Data: The data reported in this paper will be shared by the lead contact upon request. All deposited data accession IDs are given in the key resources table.
• Code: This study did not result in any development of original code.
• Any additional information required to reanalyze the data reported in this work paper is available from the lead contact upon request.

## Acknowledgments

This research was supported by the 10.13039/501100012166National Key Research and Development Program of China (2021YFC2400600/2021YFC2400605) and the 10.13039/501100001809National Natural Science Foundation of China (grant no. 82272773, 82404064, 82373177, and 82172366). We are grateful to Professor Bing Li (Shanghai Jiao Tong University School of Medicine, China) for his valuable suggestions and feedback and to Professor Dan Ye (Fudan University, China) for her insightful advice and assistance. We also thank Weixi Chen (SeekGene, China), Jun Zhang (SeekGene, China), and Chen Chen (Benagen, China) for their technical support with sequencing. Additionally, we appreciate Mingxi Li (Apeiron Therapeutics, China) and Professor Tianlong Li (Harbin Institute of Technology, China) for their contributions to this research.

## Author contributions

Conceptualization, Y.H., H. Mu, W.S., J.W., and Y.J.; methodology, H. Mu, Q.Z., Y.T., H. Meng, Z.L., X.H., and J.W.; validation, Y.H., H. Mu, and J.W.; formal analysis, H. Mu, Q.Z., D.Z., J.W., Y.T., and W.Z.; investigation, H. Mu; resources, D.Z., H.W., J.S., M.S., W.S., Y.H., H.H., J.H., M.Y., J.L., Y.Y., R.T., and Z.C.; data curation, H. Mu, Q.Z., D.Z., J.W., and Y.T.; writing – original draft, H. Mu and J.W.; writing – review and editing, all authors; visualization, H. Mu; supervision, H. Mu, Y.H., and J.W.; project administration, Z.W., Y.L., T.Z., J.X., and F.T.; funding acquisition, Z.C., H. Mu, W.S., and L.Y.

## Declaration of interests

The authors declare no competing interests.

## STAR★Methods

### Key resources table

**Table:** 

REAGENT or RESOURCES	SOURCE	IDENTIFIER
**Antibodies**		

anti-MAT2A antibody	Sigma-Aldrich	Cat#HPA043028; RRID:AB_10960707
anti-MTAP antibody	Invitrogen	Cat#PA5-22000; RRID:AB_11152677
anti-CD8A antibody	Cell Signaling Technology	Cat#85336; RRID:AB_2800052
anti-PD-L1 antibody	Cell Signaling Technology	Cat#13684; RRID:AB_2687655
anti-IKZF1 antibody	Cell Signaling Technology	Cat#14859; RRID:AB_2744523
anti-γH2Ax antibody	Cell Signaling Technology	Cat#9718; RRID:AB_2118009
anti-GAPDH antibody	Abcam	Cat#ab181602; RRID:AB_2630358
anti-beta Actin antibody	Abcam	Cat#ab8226; RRID:AB_306371
Phalloidin-iFluor 488 reagen	Abcam	Cat#ab176753; RRID:AB_3076589
HRP-conjugated goat anti-rabbit IgG	Signalway Antibody	Cat#L3042
APC anti-mouse CD45 antibody	BioLegend	Cat#103112; RRID:AB_312977
HRP-conjugated goat anti-mouse IgG	Signalway Antibody	Cat#101
PCP anti-mouse CD8 antibody	BioLegend	Cat#100731; RRID:AB_893423
APC anti-mouse CD45 antibody	BioLegend	Cat#103112; RRID:AB_312977
anti-mouse CD28 monoclonal antibody	eBioscience	Cat#14-0289-82, Clone CD28.2; RRID:AB_467194
anti-mosue CD3 monoclonal antibody	eBioscience	Cat#14-0037-82, Clone OKT3; RRID:AB_467057
anti-mouse CD28 monoclonal antibody	eBioscience	Cat#14-0289-82, Clone CD28.2; RRID:AB_467194
InVivoMAb anti-mouse PD-L1 antibody	BioXCell	Cat#BE0101, Clone 10F.9G2; RRID:AB_10949073
InVivoMAb anti-mouse PD-1 antibody	BioXCell	Cat#BE0273, Clone 29F.1A12; RRID:AB_2687796
InVivoMAb rat IgG2b isotype control	BioXCell	Cat#BE0090; RRID:AB_1107780
Alexa Fluor Plus 555	Invitrogen	Cat#A32732; RRID:AB_2633281

**Chemicals, peptides, and recombinant proteins**		

DMEM high Glucose	Gibco	Cat#11965092
DMEM/F-12	Gibco	Cat#11320033
McCoy’s 5a medium	Gibco	Cat#16600082
Fetal bovine serum	Wisent	Cat#080-150
Opti-MEM	Gibco	Cat#31985070
Phophate-Buffered Saline	Thermo Fisher Scientific	Cat#J61196.AP
Physiological saline	Shanghai yuanye Bio-Technology Co., Ltd	Cat#R21479
Tris-buffered saline	Sangon Biotech	Cat#A510025
Red Blood Cell Lysis Solution	Thermo Fisher Scientific	Cat#00-4333
Trypsin-EDTA	Thermo Fisher Scientific	Cat#15400054
Tween 20	Sangon Biotech	Cat#A100777
DNase I	Sigma-Aldrich	Cat#EN0521
RIPA lysis buffer	Beyotime	Cat#P0013B
Proteinase K	MedChemExpress	Cat#HY-108717
Triton X-100	Sangon Biotech	Cat#A600198
DAPI	Beyotime	Cat#C1002
Trypan blue	Thermo Fisher Scientific	Cat#15250061
Crystal violet staining solution	Beyotime	Cat#C0121
Puromycin	MedChemExpress	Cat#HY-B1743A
PF-9366	MedChemExpress	Cat#HY-107778
SCR6639	This study (Jiangsu Simecre Pharmaceutical Co. LTD.)	Patent: WO/2021/139775
L-Methionine	Sigma-Aldrich	#M9625-5G
Adenosine 5′-triphosphate disodium salt hydrate (ATP)	Sigma-Aldrich	#A7699-1G
Potassium chloride solution (KCl)	Sigma-Aldrich	#60142-500ML-F
Trizma® hydrochloride solution (Tris)	Sigma-Aldrich	#T2663-1L
Magnesium chloride solution (MgCl_2_)	Sigma-Aldrich	#M1028
EDTA (0.5 M), pH 8.0, RNase-free	Invitrogen	#AM9260G
Bovine Serum Albumin (BSA)	Sangon Biotech	#A500023-0100
Phosphate Assay Kit - PiColorLockTM	Abcam	#ab270004
MAT2A protein	Pharmaron Inc.	This study
MAT2A inhibitor screening assay kit	BPS bioscience	Cat#71402
Polybrene	MedChemExpress	Cat#HY-112735
PEI transfection reagent	Polyseciences	Cat#26406
B-zero vector	TransGen	Cat#CTB501-02
Nylon cell strainer (40μm)	Corning	Cat#352340
Polyvinylidene fluoride (0.45μm)	Millipore	Cat#IPFL00010
High Sensitivity DNA Chip	Agilent	Cat#5067-4626
Methionine-restricted mouse feed (0.12% methionine)	Xietong Pharmaceutical Bio-engineering Co., Ltd.	Gao X et al.^37^
Normal mouse feed (0.86% methionine)	Xietong Pharmaceutical Bio-engineering Co., Ltd.	Gao X et al.^37^
Glass bottom culture dishes	NEST	Cat#801001
100mm Culture Dishes	Corning	Cat#430167
60mm Culture Dishes	Corning	Cat#430166
6-well plates	Corning	Cat#3516
96-well plates	Corning	Cat#3798
384-well plates	Corning	Cat#3680
Methanol	CNW Technologies	Cat#67-56-1
Acetonitrile	CNW Technologies	Cat#75-05-8
Ammonium acetate	Sigma-Aldrich	Cat#631-61-8
Ammonium hydroxide	Fisher Chemical	Cat#1336-21-6

**Critical commercial assays**		

Chromium Next GEM Single Cell 3ʹ Kit v3.1	10x Genomics	Cat#PN-1000268
SeekOne DD Single Cell 3′ Transcriptome kit	SeekGene	N/A
RNeasy Mini Kit	QIAGEN	Cat#74104
RNA Nano 6000 Assay Kit	Agilent	Cat#5067-1511
HiSeq PE Cluster Kit v4-cBot-HS	Illumina	Cat#PE-401-4001
SuperSignal_TM_ West Femto Maximum Sensitivity Substrate	Thermo Fisher Scientific	Cat#34094
NEBNext® Ultra™ RNA Library Prep Kit for Illumina®	NEB	Cat#E7530
Qubit_TM_ dsDNA HS Assay Kit	Thermo Fisher Scientific	Cat#Q32851
Cell-Light EdU Apollo567 *In Vitro* Image Kit	Ribobio	Cat#C10310
Cell Counting Kit-8	Dojindo	Cat#CK04
EasySep™ Mouse Pan-Naïve T cell Isolation Kit	Stemcell	Cat#19848a
Pierce BCA Protein Quantification Kit	Thermo Fisher Scientific	Cat#23325

**Deposited data**		

Single-cell RNA-seq of Osteosarcoma	Gene Expression Omnibus	GEO: GSE152048
Single-cell RNA-seq of Osteosarcoma	Gene Expression Omnibus	GEO: GSE162454
Transcriptomic of Shanghai General Hospital Osteosarcoma Cohort	National Genomics Data Center - Genome Sequence Archive for Human	GSA: HRA003260
Methylation Profiling by Array of Shanghai General Hospital Osteosarcoma Cohort	National Genomics Data Center - OMIX	OMIX: OMIX002042
Transcriptomic of Osteosarcoma	Gene Expression Omnibus	GEO: GSE42352
GDC TARGET-OS	National Cancer Institute	TARGET’s Study of Osteosarcoma - NCI (cancer.gov)
Single-cell RNA-seq of Osteosarcoma	National Genomics Data Center - OMIX (This study)	OMIX: OMIX007614
Bulk RNA-seq of DuNN Mouse Models *in vivo*	National Genomics Data Center - Genome Sequence Archive (This study)	GSA: CRA021534
Bulk RNA-seq of 143B *in vitro*	National Genomics Data Center - Genome Sequence Archive for Human (This study)	GSA: HRA009828
Mouse Gut Fecal Metabolomics	MetaboLights (This study)	MetaboLights: MTBLS11985

**Experimental models: Cell lines**		

HOS	ATCC	Cat#CRL-1543 CVCL_0312
HOS/MNNG	ATCC	Cat#CRL-1547 CVCL_0439 Cl#5
143B	ATCC	Cat#CRL-8303 CVCL_2270
SJSA-1	ATCC	Cat#CRL-2098 CVCL_1697
U2OS	ATCC	Cat#HTB-96 CVCL_0042
DuNN	Dunn Lab	CVCL_W629
HCT116-MTAP-KO-11B1	Kyinno Bio	Cat#KC-1405
HEK-293T	ATCC	Cat#CRL-3216 CVCL_0063
Human Mesenchymal Stem Cell (hMSC) Cell line	This study	N/A

**Experimental models: Organisms/strains**		

C3H/HeNCrl	Charlers River	RRID: IMSR_CRL:025
BALB/cJGpt-Foxn1^nu^/Gpt	Charlers River	RRID: IMSR_GPT: D000521

**Oligonucleotides**		

See Table S8 for oligonucleotides	This study	N/A

**Software and algorithms**		

GraphPad Prism (version 8)	GraphPad	RRID:SCR_002798
RStudio	posit	RRID:SCR_000432
ImageJ	ImageJ	RRID:SCR_003070
R software (version 4.2.0)	The R Foundation	RRID:SCR_001905
FlowJo (version 10)	FlowJo	RRID:SCR_008520
Leica Application Suite X (LAS X)	Leica	RRID:SCR_013673
GRCh38 genome	NIH	Homo sapiens genome assembly GRCh38 - NCBI - NLM (nih.gov)
mm10 genome	NIH	Mus musculus genome assembly GRCm38 - NCBI - NLM (nih.gov)
bcl2fastq2 Conversion Software (version 2.20)	Illumina	RRID:SCR_015058
FastQC (version 0.11.9)	Babraham Bioinformatics	RRID:SCR_014583
Cell Ranger (version 3.0.1)	10x Genomics	RRID:SCR_017344
DoubletFinder package (version 2.0.2)	McGinnis et al.^49^	RRID:SCR_018771
Harmony package	Korsunsky et al.^50^	RRID:SCR_022206
clusterProfiler (version 3.14.0)	Yu et al.^51^	RRID:SCR_016884
iTALK package	doi.org/10.1101/507871	N/A
inferCNV package (version 1.12.0)	10.18129/B9.bioc.infercnv	N/A
biomaRt package (version 2.42.1)	Durinck et al.^52^	RRID:SCR_019214
STAR (version 2.7.6a)	Dobin et al.^53^	RRID:SCR_004463
DESeq2	https://doi.org/10.1186/s13059-014-0550-8	RRID:SCR_015687
CIBERSORTx	Newman et al.^54^	RRID:SCR_016955
Gene Set Enrichment Analyiss (GSEA, version 4.1.0)	Subramanian et al.^55^	RRID:SCR_003199
ALGGEN-PROMO	Messeguer et al.^56^	RRID:SCR_016926
Gene Expression Profiling Interactive Analysis (GEPIA)	Tang et al.^57^	RRID:SCR_018294
Xena	University of California at Santa Cruz	RRID:SCR_018938
R2: Genomics Analysis and Visualization Platform	Amsterdam UMC	https://hgserver1.amc.nl/cgi-bin/r2/main.cgi?open_page=login
BioRender	BioRender	RRID:SCR_018361
CaseViewer (version 2.4)	3DHISTECH Ltd.	RRID:SCR_017654

**Other**		

Illumina NovaSeq 6000 Sequencing System	Illumina	RRID:SCR_016387
Invitrogen Countess II Automated Cell Counter	Invitrogen	RRID:SCR_025370
Agilent 2100 Bioanalyzer Instrument	Agilent Technologies	RRID:SCR_018043
DNBSEQ-T7	BGI	RRID:SCR_017981
SpectraMax M3 Microplate Reader	Molecular Devices	Cat#14327
PowerPac HV Power Supply	Bio-Rad	Cat#1645056
Amersham Imager 600	GE Healthcare	RRID:SCR_021853
Leica SP8 LIGHTNING confocal microscope	Leica	RRID:SCR_018169
BD LSRFortessa Cell Analyzer	BD Biosciences	RRID:SCR_018655
10X Genomics Chromium Controller Genetic Analyzer	10x Genomics	RRID:SCR_019326
SeekOne DD Chip platform	SeekGene	N/A
Vanquish™ Refractive Index Detector	Thermo Fisher Scientific	Cat#VC-D60-A01
Orbitrap Exploris™ 120 Mass Spectrometer	Thermo Fisher Scientific	Cat#BRE725531
Fresco™ 17 Microcentrifuge	Thermo Fisher Scientific	Cat#75002402

### Experimental model and study participant details

#### Study cohort and collection of clinical human samples

Our study was approved by the Institutional Research Ethics Committee of Shanghai General Hospital and Shanghai Sixth Hospital, Shanghai Jiao Tong University School of Medicine. This study includes a total of 20 osteosarcoma patients, comprising 5 females and 15 males, aged between 8 and 70 years (mean ± SD: 30.85 ± 17.53). Informed consent was obtained from all patients. Detailed information is provided in Tables S1 and S2.

The retrospective following-up cohort in our study included OS patients (*n* = 13) at Shanghai General Hospital and Shanghai Sixth Hospital (Figure 1A). The inclusion criteria comprised advanced osteosarcoma patients who received off-label immune checkpoint therapy (ICT) following failure of multiple lines of treatment. The exclusion criteria included osteosarcoma patients who did not undergo ICT, those lost to follow-up, and patients whose treatment was discontinued for any reason or who voluntarily withdrew from treatment.

The single-cell RNA sequencing (scRNA-seq) data used in this study were derived from two sources (Figure 1B). The first set comes from patient data collected at Shanghai General Hospital (*n* = 8), and the second set comprises previously published data (*n* = 4), including one individual from Liu et al. (GSE162454)^58^ and three individuals from Zhou Y et al. (GSE152048).^59^ The samples were categorized into MTAP-deleted (DEL) and MTAP wild-type (WT) based on their MTAP condition. Samples from Shanghai General Hospital (*n* = 8) were collected in the core of the tumor resections. The patient data from Shanghai General Hospital were determined by genomic (NGS) sequencing of the same tissue at the same sampling time (DEL = 4, WT = 4). This was further validated using inferCNV analysis from subsequent scRNA-seq data to detect CNV.^60^ For the published data, samples for the DEL group (*n* = 2) were selected based on previously published inferCNV-detected CNV data, while samples for the WT group (*n* = 2) were randomly chosen from all other data in GSE162454 and GSE152048. This ensured that the sample sizes of both groups remained consistent.

The specimens of SGH-OS cohort in this study (*n* = 50) were extend from our previous study (GSA: HRA003260, OMIX002042),^16^ including clinical information, copy number array data for genomic copy number variation, bulk RNA-seq data for transcriptomic and tissue micro-array (TMA) (this study) for protein expression and histopathology (Figure 1C). The TMAs were created using tumor-containing paraffin blocks selected based on pathologist observation of matched tissue, significantly reducing the likelihood of sample collection or processing bias (this study paired to SGH-OS) (Table S7). Samples of SGH-OS including in this study were collected in the core of the tumor resections. Pathological diagnoses of all the OS patients admitted in Shanghai General Hospital were independently examined by three pathologists. Clinical information and gene expression related to patient survival (GSE42352) for survival analysis were downloaded from Gene Expression Omnibus (GEO).

#### Cell line culture

The human OS cell lines HOS, HOS/MNNG, 143B, SJSA-1 and U2OS, the mouse OS cell line DuNN and the human kidney cell line HEK-293T were cultured in high glucose Dulbecco’s Modified Eagle Medium (DMEM) supplemented with 10% fetal bovine serum. Cell line authentication was performed on cells that used for *in vitro* and *in vivo* studies using Short Tandem Repeat (STR) DNA profiling. Cell lines are regularly tested for mycoplasma contamination. All cell lines were preserved at Shanghai Bone Tumor Institute (Shanghai, China). The human mesenchymal stem cell (hMSC) cell line was cultured in Dulbecco’s Modified Eagle Medium/Nutrient Mixture F-12 (DMEM/F-12) medium supplemented with 10% fetal bovine serum (FBS). HCT116-MTAP-KO-11B1 cells were cultured in McCoy’s 5A medium supplemented with 10% fetal bovine serum and 1% penicillin–streptomycin. All cell lines were cultured at 37°C in a 5% CO_2_ incubator.

#### Animal experiments

All animal experiments were performed by following the protocols in accordance with the guidelines of Laboratory Animal Center of Shanghai General Hospital. The Clinical Center Laboratory Animal Welfare & Ethics Committee of Shanghai General Hospital, Shanghai Jiao Tong University School of Medicine, approved all animal protocols used in this study. C3H mice (Strain: C3H/HeNCrl; Charlers river, USA/China) and Nude mice (Strain: BALB/cJGpt-foxn1nu/Gpt; Charlers river, USA/China) were used in this study. All the mouse models were female and 6-8-week year old.

All mice were maintained under SPF conditions in a controlled environment of 20°C–22°C, with a 12/12h light/dark cycle, 50–70% humidity. The OS mouse tibial-orthotopic xenograft was developed by intramedullary injection with OS cells (5×10^5^ cells in 25ul PBS supplemented with 10% FBS) into the marrow space of the proximal tibial of C3H or Nude mice with a 27-gauge needle, as our previous study.^61^ OS progression in the mice was monitored by measuring the tumor volume, calculated by the following equation: Volume = Length×Width^2^/2, using a caliper. The tumor burden was monitored by following the tumor volume and the maximal tumor size is 2 cm, which was permitted by the Clinical Center Laboratory Animal Welfare & Ethics Committee of Shanghai General Hospital, Shanghai Jiao Tong University School of Medicine. The maximal tumor size was confirmed to be not exceeded in our study and the survival curves were produced according to the survival of mice and the time to reach maximum tumor size. Samples was from the core of tumor resections.

For methionine diet restriction studies, the mice were evaluated by monitoring tumor volume to quantify OS burden before for randomization and in the progress of drug treatment for efficacy evaluation. 14 days prior to constructing the tibia orthotopic model of osteosarcoma in mice, the methionine restriction group was placed on a methionine-restricted diet (containing 0.12% methionine), while non-restriction group received a normal diet (containing 0.86% methionine).^37^ If combined with immunotherapy, anti-PD-1 monoclonal antibody (i.p.,5 mg/kg at Day7, Day9, Day10 and Day15) would be used. For MAT2A inhibition combination studies, the mice were evaluated by monitoring tumor volume to quantify OS burden before for randomization and in the progress of drug treatment for efficacy evaluation. Mice were treated with either vehicle (Physiological saline) and isotype IgG2b (Control group), anti-PD-L1 monoclonal antibody (i.p.,10 mg/kg at Day7, 5 mg/kg at Day12, 2.5 mg/kg biw Day14 to Day 42; ICT alone group), SCR6639 (i.g., solved in 10% NMP+10% Solutol+80% physiological saline, 3 mg/kg bid Day14 to Day42; SCR6639 alone group), or combined anti-PD-L1 monoclonal antibody and SCR6639 (Combined group).

### Method details

#### Preparation and analysis of single-cell RNA sequencing (scRNA-seq)

Tissue transportion and dissociation were according to Zhou Y et al.^59^ and Liu et al.^58^ Tumor tissues transported under 4°C and were transported in sterile culture dish with 10mL 1X Phophate-Buffered Saline (PBS; Thermo Fisher Scientific, USA) on ice to remove the residual tissue storage solution. Fat tissue and visible blood vessels were removed before process and then cut into small pieces. Dissociation enzyme 0.25% Trypsin (Thermo Fisher Scientific) and 10ug/ml DNase I (Sigma-Aldrich) dissolved in PBS with 5% FBS were used to digest the tissues. Tumor tissues of OS were dissociated at 37°C with a shaking speed of 50 rpm for about 40 min. The dissociated cells were repeatedly collected at an interval of 20 min to increase cell yield and viability. Cell suspensions were filtered using a 40um nylon cell strainer (Corning, USA) and red blood cells were removed by 1× Read Blood Cell Lysis Solution (Thermo Fisher Scientific, USA). Dissociated cells were washed with PBS containing 2% FBS twice. Cells were stained with 0.4% Trypan blue (Thermo Fisher Scientific, USA) to check the cell viability on Countess II Automated Cell Counter (Thermo Fisher Scientific, USA). Freshly prepared cell suspensions were performed immediately according to the manufacturer’s protocol of 10X Chromium 3′ v3.1 kit (10X Genomics, USA) or SeekOne DD Single Cell 3′ Transcriptome kit (SeekGene, Beijing, China). Samples prepared by 10X Chromium 3’ v3.1 kit were used 10X Genomics Chromium Controller Genetic Analyzer for sequencing library establishment and prepared by SeekOne DD Single Cell 3′ Transcriptome kit were used SeekOne DD Chip platform for sequencing library establishment. Sequencing libraries were quantified using a High Sensitivity DNA Chip (Agilent) on a Bioanalyzer 2100 and the Qubit High Sensitivity DNA Assay (Thermo Fisher Scientific). The libraries were sequenced on a NovaSeq6000 platform (Illumina, USA). The lung metastasis tumor lesions were prepared in Shanghai Biotechnology Corporation (SBC; Shanghai, China), using 10X Genomics Chromium Controller Genetic Analyzer (10X Genomics, USA) and the primary tumor lesions were prepared in SeekGene Corporation (SeekGene, Beijing, China), using SeekOne DD Chip platform (SeekGene, Beijing, China). The integration of single-cell sequencing data using various techniques, as employed in this study, has been proven feasible in previous research.^62^

For quality control and normalization. Raw sequencing reads were transformed into FASTQ files with Illumina bcl2fastq2 Conversion Software v2.20 and quality checked with FastQC software v0.11.9 at https://www.bioinformatics.babraham.ac.uk/projects/fastqc/. Standard pipelines of Cell Ranger (version 3.0.1) were used to do sequence processing, alignment to GRCh38 genome with default parameters (https://support.10xgenomics.com/single-cell-gene-expression/software/pipelines/latest/using/running-pipes-overview) by STAR algorithm. Gene-barcode matrices were generated for each individual sample by counting unique molecular identifiers (UMIs) and filtering non-cell associated barcodes.

For dimension reduction and cell-clustering analysis. The raw output data were processed with the Seurat package (version 4.0, http://satijalab.org/seurat/) in R software (version 4.2.0) for each individual sample. The Seurat object with gene expression data from individual samples was processed with the Read 10× function. We filtered out the cells with no. of expressed genes less than 300 genes or more than 6000, or the percent of mitochondrial genes over 50% of total expressed genes. Filtered data were log normalized using default scaling factor. The top 2000 highly variable genes (HVGs) from the normalized expression matrix were identified, centered and scaled before we performed the principal component analysis (PCA) based on these HVGs. Integration and analysis of single-cell data were performed using canonical correlation analysis (CCA) method in the Seurat package. The clustering analysis was performed based on the integrated joint embedding data. The identified clusters were visualized on the 2D map produced with the UMAP method. We also removed the specific cells of lung tissue. A total of 100,416 filtered cells were used for further bioinformatic analysis. To annotate the cell clusters, differentially expressed genes (DEGs) with high discrimination abilities between the groups were identified in Seurat using the default non-parametric Wilcoxon rank-sum test with Bonferroni correction. The cell groups were annotated based on the DEGs. The analysis of cell receptor-ligand pairs utilized the iTALK package in R (https://github.com/Coolgenome/iTALK).^63^

For single-cell copy-number variation (CNV) analysis. Each cell in the samples was estimated with the inferCNV package of R (version 1.12.0, https://github.com/broadinstitute/inferCNV/wiki).^60^ The immune cells (T cells) were applied as the reference. The inferCNV analysis was performed with parameters including “denoise”, default hidden markov model (HMM) settings, and a value of 0.1 for “cut-off”. To reduce the false positive CNV calls, the default Bayesian latent mixture model was implemented to identify the posterior probabilities of the CNV alterations in each cell with the default value of 0.5 as the threshold. To infer the clonal single-cell CNV changes, the “subcluster: method was applied to infer the subcluster cells based on the CNV values generated by HMM.

#### Preparation and analysis of bulk RNA sequencing (RNA-seq)

Total RNA was extracted using TRIzol reagent (Invitrogen) according to the manufacturer’s instructions. The integrity and purity of RNA were assessed by agarose gel electrophoresis and Nanodrop spectrophotometer (Thermo Fisher Scientific). RNA purity of specimens was checked using the kaiaoK5500 Spectrophotometer (Kaiao, China). RNA integrity and concentration was assessed using the RNA Nano 6000 Assay Kit of the Bioanalyzer 2100 system (Agilent Technologies, USA). A total amount of 2ug RNA per sample was used as input material for the RNA sample preparations. Sequencing libraries were generated using NEBNext Ultra RNA Library Prep Kit for Illumina (NEB, USA) according to the manufacturer’s protocol and index codes were added to attribute sequences to each sample. The clustering of the index-coded samples was performed on a cBot cluster generation system using HiSeq PE Cluster Kit v4-cBot-HS (Illumina, USA) according to the manufacturer’s instructions. After cluster generation, the libraries were sequenced on DNBSEQ-T7 platform (BGI, China) in Wuhan Benagen Technology Co., Ltd. (Wuhan, China) and 150 bp paired-end reads were generated. The raw data were first processed with FastQC to filter out adapters and low-quality sequences. Pair-end reads were aligned to human GRCh38 genome or mouse mm10 genome using STAR (v2.7.6a). Reads with good mapping quality (MAPQ >30) that aligned to genomic exons were counted using featureCounts (GRCh38 or mm10 Ensembl 93) to generate a table with counts for each gene. Differential gene expression analysis was performed using the R package DESeq2 using the IfcShrink function. Genes with fold-change ≥2.00, probability ≥0.80 and false discovery rate *p* value (FDR) < 0.05 were considered significantly differentially expressed. The transform between human genome and mouse genome was performed using the R package BioMart.

Gene Ontology (GO; http://geneontology.org/) enrichment analyses for differentially expressed genes were performed using the R package clusterProfiler v3.8. Gene set enrichment analysis (GSEA; http://www.gsea-msigdb.org/gsea/) was performed on list of genes ranked from high to low DESeq2 estimated fold-change using the GSEAPreRanked function with enrichment statistic classic, 1000 permutations and normalized *p* value <0.05. Related gene sets were downloaded from Molecular Signatures Database (MSigDB, https://www.gsea-msigdb.org/gsea/msigdb/). Gene co-expression analysis with PD-L1 in SGH-OS and TARGET-OS database was calculated according to the Spearman’s correlation analysis and genes co-expressed with PD-L1 were performed using the online tool PROMO (http://alggen.lsi.upc.es/cgi-bin/promo_v3/promo/) to predict transcriptional factors.

For bulk RNA-seq deconvolution, to establish the proportions of immune cells in our SGH-OS cohort (*n* = 50) from bulk RNA-seq, the online tool CIBERSORTx^27^^,^^28^^,^^29^ was used to estimate cell-type proportions. The normalized gene expression data was uploaded to the web portal (https://cibersortx.stanford.edu/) using the reference signature matrix according to our scRNA-seq data with permutations of 1000. After analyzing the deconvoluted data, samples that could not be successfully deconvoluted (e.g., *p* value >0.05) were excluded. A total of 47 patients' bulk RNA-seq deconvolution data were included for subsequent analysis.

#### Mouse gut fecal metabolomics utilizing LC-MS/MS analyses

25 mg of sample was weighted to an EP tube, and 500 μL extract solution (methanol: acetonitrile: water = 2: 2: 1, with isotopically-labelled internal standard mixture) was added. Then the samples were homogenized at 35 Hz for 4 min and sonicated for 5 min in ice-water bath. The homogenization and sonication cycle were repeated for 3 times. Then the samples were incubated for 1 h at −40°C and centrifuged at 12000 rpm (RCF = 13800(×g), R = 8.6cm) for 15 min at 4°C. The resulting supernatant was transferred to a fresh glass vial for analysis. The quality control (QC) sample was prepared by mixing an equal aliquot of the supernatants from all of the samples. LC-MS/MS analyses were performed using an UHPLC system (Vanquish, Thermo Fisher Scientific) with a Waters BEH Amide column (2.1 mm × 50 mm, 1.7 μm) coupled to Orbitrap Exploris 120 mass spectrometer (Orbitrap MS, Thermo). The mobile phase consisted of 25 mmol/L ammonium acetate and 25 mmol/L ammonia hydroxide in water(pH = 9.75)(A) and acetonitrile (B). The auto-sampler temperature was 4°C, and the injection volume was 2 μL. The Orbitrap Exploris 120 mass spectrometer was used for its ability to acquire MS/MS spectra on information-dependent acquisition (IDA) mode in the control of the acquisition software (Xcalibur, Thermo). In this mode, the acquisition software continuously evaluates the full scan MS spectrum. The ESI source conditions were set as following: sheath gas flow rate as 50 Arb, Aux gas flow rate as 15 Arb, capillary temperature 320°C, full MS resolution as 60000, MS/MS resolution as 30000 collision energy as 20/30/40 in NCE mode, spray Voltage as 3 kV (positive) or −3 kV (negative), respectively. The raw data were converted to the mzXML format using ProteoWizard and processed with an in-house program, which was developed using R and based on XCMS, for peak detection, extraction, alignment, and integration. Then an in-house MS2 database (BiotreeDB) was applied in metabolite annotation. The cutoff for annotation was set at 0.3.

#### Biochemical assay for MAT2A activity

Compounds (e.g., SCR6639) were dissolved in DMSO and diluted to a final concentration of 10 μM with a 3-fold serial dilution. Subsequently, 80 nL of each dilution was transferred to a 384-well plate. The assay buffer was prepared using 50 mM Tris, 50 mM KCl, 15 mM MgCl_2_, 100 μM EDTA, and 0.005% BSA. MAT2A protein was diluted to a final concentration of 4 μg/mL in the assay buffer. To each well of the 384-well plate, 40 μL of 2× MAT2A solution was added, followed by centrifugation at 1000 rpm for 1 min and incubation at room temperature (RT) for 120 min. L-methionine and ATP were diluted in the assay buffer to final concentrations of 200 μM and 400 μM, respectively. Subsequently, 40 μL of the 2× L-methionine and ATP solution was added to initiate the reaction. The plate was centrifuged at 1000 rpm for 1 min and incubated at RT for 90 min. According to the manufacturer’s instructions, the PiColorLockTM reaction catalyst and PiColorLockTM buffer were mixed at a 1:100 ratio. Then, 20 μL of the mixture was added to each well and shaken for 30 s. Next, 8 μL of the stabilization reagent was added and shaken for an additional 30 s. After incubating at RT for 30 min, the signal values were detected. % compound inhibition was calculated, and the IC_50_ values of the compounds were determined through curve fitting.

Detection of Intracellular S-Adenosylmethionine (SAM) Levels.

HCT116-MTAP-KO-11B1 cells were cultured in McCoy’s 5A medium supplemented with 10% fetal bovine serum and 1% penicillin–streptomycin at 37°C in a 5% CO_2_ incubator. Only logarithmically growing cells were utilized for experiments. The impact of compounds on SAM levels in HCT116-MTAP-KO-11B1 cell lines was assessed using LC-MS/MS. Cell density was adjusted to 50,000 cells per well and seeded into 96-well plates, followed by overnight incubation under the same conditions (37°C, 5% CO_2_). Compounds were dissolved in DMSO, subsequently diluted with DMSO and culture medium, and transferred to the cell plates to achieve a final concentration of 10 μM with a 3-fold serial dilution. The plates were then incubated at 37°C in a 5% CO_2_ environment for an additional 6 h. After incubation, the supernatant was aspirated, and cells were washed once with PBS. Ice-cold acetic acid was added to lyse the cells. The lysate was processed and analyzed by LC-MS/MS to quantify SAM concentrations. % Compound inhibition was calculated, and the IC_50_ values of the compounds were determined through curve fitting.

#### Cell viability assay and colony formation

To assay cell growth, OS cells were washed twice with PBS and plated onto 60mm cell culture dishes at a density of 3000 cells per dish. OS cells were treated with or without drugs for 1 week, and the cell colonies were stained using crystal violet staining solution (Beyotime, China) according to the manufacturer’s instructions. To assay cell viability, OS cells were plated onto 96-well plates at a density of 3000 cells per well. OS cells were treated with or without drugs for 3 days, and cell viabilities were then measured by using a Cell Counting Kit-8 (CCK-8; #CK04, Dojindo, Japan) detected using SpectraMax M3 Microplate Reader (Molecular Devices, USA).

#### Immunohistochemical (IHC), immunofluorescence and histopathology staining

For IHC and histopathology staining. IHC staining was performed on representative tissue sections from formalin-fixed and paraffin-embedded tissue blocks from human OS TMA and mice tumor xenografts using the mentioned antibodies at the indicated concentrations. Hematoxylin & eosin (H&E) staining of human OS TMA and mice tumor xenografts was performed as our previous study.^64^

For immunofluorescence staining. Cells on the glass bottom culture dishes (#801001, NEST, China) were fixed in 4% parafomaldehyde (diluted the 32% paraformaldehyde in PBS) for 10 min at room temperature. Cells were washed three times for 5 min with 200mM glycine containing PBS, followed by permeabilization with 0.3% Triton X-100 in PBS for 15 min. After blocking with 5% bovine serum albumin (BSA) in PBS for 1h, cells were incubated with mentioned primary antibody (γH2Ax) diluted in a 5% BSA in PBS solution overnight at 4°C. After washing four times with PBS, cells were incubated with Alexa Fluor Plus 555 (1:500; #A32732, Invitrogen, USA) secondary antibody for 1h at room temperature and washed three times with PBS. Cell skeletons were stained with phalloidin-iFluor 488 reagent for 20 min followed by washed with PBS three times. Cell nuclei were then counterstained with DAPI (Beyotime, China) for 5 min. Cells were washed two more times in PBS before imagine. 5′-ethynyl, 2′-deoxyuridine (EdU) incorporation analysis was performed using Cell-Light EdU Apollo567 *In Vitro* Image Kit (#C10310, Ribobio, China) according to the manufacturer’s protocol. Images were acquired using Leica TCS SP8 Laser Scanning Confocal Microscope (Leica, USA) and were processed by Leica Application Suite X (LAS X; Leica, USA).

#### General cloning and plasmid constructs

Constitutive expression of shRNA hairpins targeting MTAP (1:5′-AGTTTACTCCTCACTACTATA-3′, 2:5′-GCGGTGAAGATTGGAATAATT-3′, 3:5′-GGACTTCGGTGCCATTCAAAG-3′) or a scramble non-targeting control (5′-TTCTCCGAACGTGTCACGT-3′) was achieved using lentiviral infection of the pGMLV vector (modified from Genomeditech, Shanghai, China), selected with puromycin (2ug/ml). DuNN^Mtap−/−^ cells were generated using the CRlSPR-Cas9 System following the manufacturer’s protocol. To construct CRlSPR/Cas9 vectors, two sgRNAs, one targeting in Exon1 (TCTCACCTTCACCGCCGTGC) and one targeting downstream of Intron1 (ATCACCCTACAAGCTGTGAA) were annealed and were cloned into pCas9 vector using Bbsl digestion to obtain pCas9-Mtap-sgR1 and pCas9-Mtap-sgR4. The cDNA of Mtap^WT^ or Mtap^G668A^ were amplified form DuNN cells via RT-PCR using primers (Mtap_F 5′-GAGCTCGAGATGGCCTCGGGCTCCGCCTG-3′, Mtap_R 5-CTCGCGGCCGCTTAATGTCTTGGTGGTAAAACGGAA-3', Mtap_G668A_mut_F 5′-ACTATGATTATTGGAAGGAGCATGA-3′, Mtap_G668A_mut_R 5′-TTCCAATAATCATAGTCGGTTGCCA-3). PCR fragments were digested with Xhol/Notl digestion and cloned into the vector to obtain pCDH-CMV-3×Flag-Mtap^WT^ puro or pCDH-CMV-3×Flag-Mtap^G668A^.

#### CRISPR/Cas9 Knock-out in DuNN cells

DuNN^Mtap−/−^ cells were generated using the CRISPR-Cas9 System following the manufacturer’s protocol. Briefly, cells were co-transfected with the two pspCas9(BB)-2A-puro plasmids containing nickase gRNA1 and gRNA4 using Lipofectamine 3000 Transfection Reagent (#L3000015, Thermo Fisher Scientific, USA) according to the manufacturer’s protocol. After transfection for 36h, cells were selected by adding 3ug/ml puromycin. After 4 days, cells were diluted into 96-well plates. After 14 days, single clones were transferred into 6-well plate and the endogenous Mtap deletion in both alleles were detected by western bloting.

#### Genomic PCR

The genomic DNA of each single clone were isolated to genotype clones. The cells were lysed in lysis buffer (75mM NaCl, 25mM EDTA, 1%SDS and 0.1ug/ul Proteinase K) and incubated for 65°C for 8 h. Then the chloroform was added and mix on the rotator for 1 h, followed by centrifugation (5500rpm, 1h at 4°C). The supernatant was collected, and the genomic DNA was precipitated with 1 volume isopronanol. After centrifugation (13000rpm, 15min, 4°C), the pellet was washed with 70% ethanol and dissolved in TE buffer (10 mM Tris-HCL and 0.1 mM EDTA pH 8.0). Genomic PCR was carried out using primers (Mtap_KO_test_F 5′-gggaggaagaggaggagccagagcc-3′, Mtap_KO_test_R 5′-aatgcagatgagttcaaaggctggg-3′) and 2×Flsh Hot Start MasterMix (#CW3007M,CWBIO, Beijing, China) with the following protocol for 30 cycles: 95°C/15s, 60°C/15s, 72°C/45s. PCR fragments were cloned into B-zero vector (#CTB501-02, TransGen, Beijing, China), and the insert fragments were sequenced by M13F primer 5′-TGTAAAACGACGGCCAGT-3’.

#### Lentivirus generation and harvesting

For shMTAP, Lentivirus production was obtained from PEI transfection reagents (#26406, Polyseciences, USA) of HEK-293T cells with co-transfection of the packaging vectors pspAX2 and pMD2.G along with the gene delivery vector. Viral supernatants were collected 72h after transfection, underwent ultracentrifugation at 20,000rpm for 25 h at 4°C to concentrate, and the virus pellets were resuspended in PBS. For infection, the viral pellets were added to cells in a dropwise manner in the presence of polybrene (10μg/ml). After 48h, medium containing the lentivirus was replaced and infected cells were selected by addition of puromycin (2ug/ml). For Mtap^WT^ or Mtap^G668A^, Lentivirus production was obtained from PEI transfection of HEK293T cells with co-transfection of the packaging vectors pspAX2 and pMD2.G along with the gene delivery vector containing Mtap^WT^ or Mtap^G668A^. Viral supernatants were collected after transfection for 48h and 72h, the virus was filtered using 0.45μm filter diam. For infection, the virus was added into plate mixed with 1 volume of fresh DMEM in the presence of polybrene (8 μg/ml). After 24h, medium containing the lentivirus was replaced and infected cells were selected by addition of puromycin (3 μg/ml).

#### Small interfering RNA (siRNA) interference experiment

On the day before transfection, 0.5–2 × 10ˆ5 cells were seeded in 400μL of antibiotic-free medium to achieve a cell density of 30%–50%. The siRNA (Genomeditech, Shanghai, China) was diluted (final concentration at 50nM) in 50μL of Opti-MEM (Gibco, USA) and gently mixed by pipetting. Separately, 1.0μL of Lipofectamine 3000 (Thermo Fisher Scientific, USA) was diluted in 50μL of Opti-MEM, gently mixed by pipetting, and allowed to stand at room temperature for 5 min. The transfection reagent and siRNA dilution were then combined to form the transfection complex, gently mixed by pipetting, and incubated at room temperature for 20 min. The transfection complex was gently added dropwise into the cell well plate and mixed by cross-pipetting. The cells were incubated at 37°C with 5%CO2 for 18h–48h, with the medium replaced with complete medium 4h–6h after transfection. The silencing efficiency of the siRNA was validated using Western blot (WB).

#### Western blot

Proteins were extracted by using radio immunoprecipitation assay (RIPA) lysis buffer (Beyotime, China) for total protein and Histone Extraction Kit (#ab113476, Abcam, USA) for histone following its manufacturer’s protocol. The extracted protein was quantified using a Pierce BCA Protein Quantification Kit (#23325, Thermo Fisher Scientific, USA) calculated using SpectraMax M3 Microplate Reader (Molecular Devices, USA). Proteins were separated by sodium dodecyl sulfate polyacrylamide gel electrophoresis (SDS-PAGE) and transferred to 0.45μm polyvinylidene fluoride (Millipore, USA) using Mini-PROTEAN Tetra Vertical Electrophoresis Cell electrophoresis chamber and Mini Trans-Blot Module for tank transfer system with PowerPac HV Power Supply (Bio-Rad, USA). The membrane was blocked using 5% nonfat dry milk in Tris-buffered saline solution containing 0.1% Tween 20 (TBS-T) for 1h at room temperature and then probed with specific primary antibodies overnight at 4°C. Subsequently, the membranes were washed with TBS-T 10 min for third times, followed by incubating with HRP-conjugated secondary antibodies for 1h at room temperature. Actin and GAPDH were used as the protein loading control. Protein signals were developed with SuperSignal West Femto Maximum Sensitivity Substrate (Thermo Fisher Scientific, USA) and imaged using chemiluminescence imaging system Amersham Imager 600 (GE Healthcare, USA) and Tanon 5200 (Tanon, China).

#### T cell co-culture and flow cytometry

Wash the culture conditions with anti-mouse CD3 antibody and incubate the isolated mouse spleen T cells with anti-mouse CD28 antibody. Add these T cells to the culture medium of adherent tumor cells for suspension co-culture. After incubating for 16–24 h, use flow cytometry (BD Biosciences, LSRFortessaTM, USA) for detection.

### Quantification and statistical analysis

Details of statistical analyses of the various experiments are described in the relevant methods section. not specified, statistical analysis was carried out using GraphPad Prism 8 software (GraphPad Software, USA). After confirming that values followed a normal distribution, two-tailed Student’s t test was applied to determine the significance of differences between two groups of independent samples. Spearman’s correlation analysis was performed to determine the correlation between two group of variables. The image was subjected to gray value analysis using ImageJ software (Thermo Fisher Scientific, USA). The Kaplan-Meier survival curves compared by two-side log rank test was performed. A *p* value <0.05 was considered statistically significant. Details of the data points shown were described in the respective figure legends. All schematic diagrams were created using BioRender (BioRender.com).

## Supplemental information

Document S1. Figures S1–S16

Table S1. Clinical cohort information of osteosarcoma patients receiving ICT treatment and follow-up, related to Figure 2

Table S2. Clinical cohort information of osteosarcoma patients derived from single-cell sequencing data, related to Figure 3

Table S3. Cell annotations for each cell subpopulation, related to Figure 3

Table S4. Proportions of each cell subpopulation, related to Figure 3

Table S5. The transcriptome grouping and matrix used for SGH-OS patients, related to Figure 4

Table S6. Proportions of each cell subpopulation in SGH-OS patients after deconvolution, related to Figure 4

Table S7. SGH-OS patients corresponding to the tissue microarray in this study, related to Figure 4

Table S8. Oligonucleotide information used in this study, related to key resources table

Document S2. Article plus supplemental information
