# Supplementary material for: Methionine intervention induces PD-L1 expression to enhance the immune checkpoint therapy response in MTAP-deleted osteosarcoma
Source: Cell Rep Med. 2025 Feb 20;6(3):101977. doi: 10.1016/j.xcrm.2025.101977 (PMC11970323; doi:10.1016/j.xcrm.2025.101977)
Supplement: Document S1. Figures S1–S16 [file mmc1.pdf]

Cell Reports Medicine, Volume *6*

Supplemental information

Methionine intervention induces PD-L1 expression to enhance the immune checkpoint therapy response in MTAP-deleted osteosarcoma

Haoran Mu, Qi Zhang, Dongqing Zuo, Jinzeng Wang, Yining Tao, Zhen Li, Xin He, Huanliang Meng, Hongsheng Wang, Jiakang Shen, Mengxiong Sun, Yafei Jiang, Weisong Zhao, Jing Han, Mengkai Yang, Zhuoying Wang, Yu Lv, Yuqin Yang, Jing Xu, Tao Zhang, Liu Yang, Jun Lin, Feng Tang, Renhong Tang, Haiyan Hu, Zhengdong Cai, Wei Sun, and Yingqi Hua

Figure S1

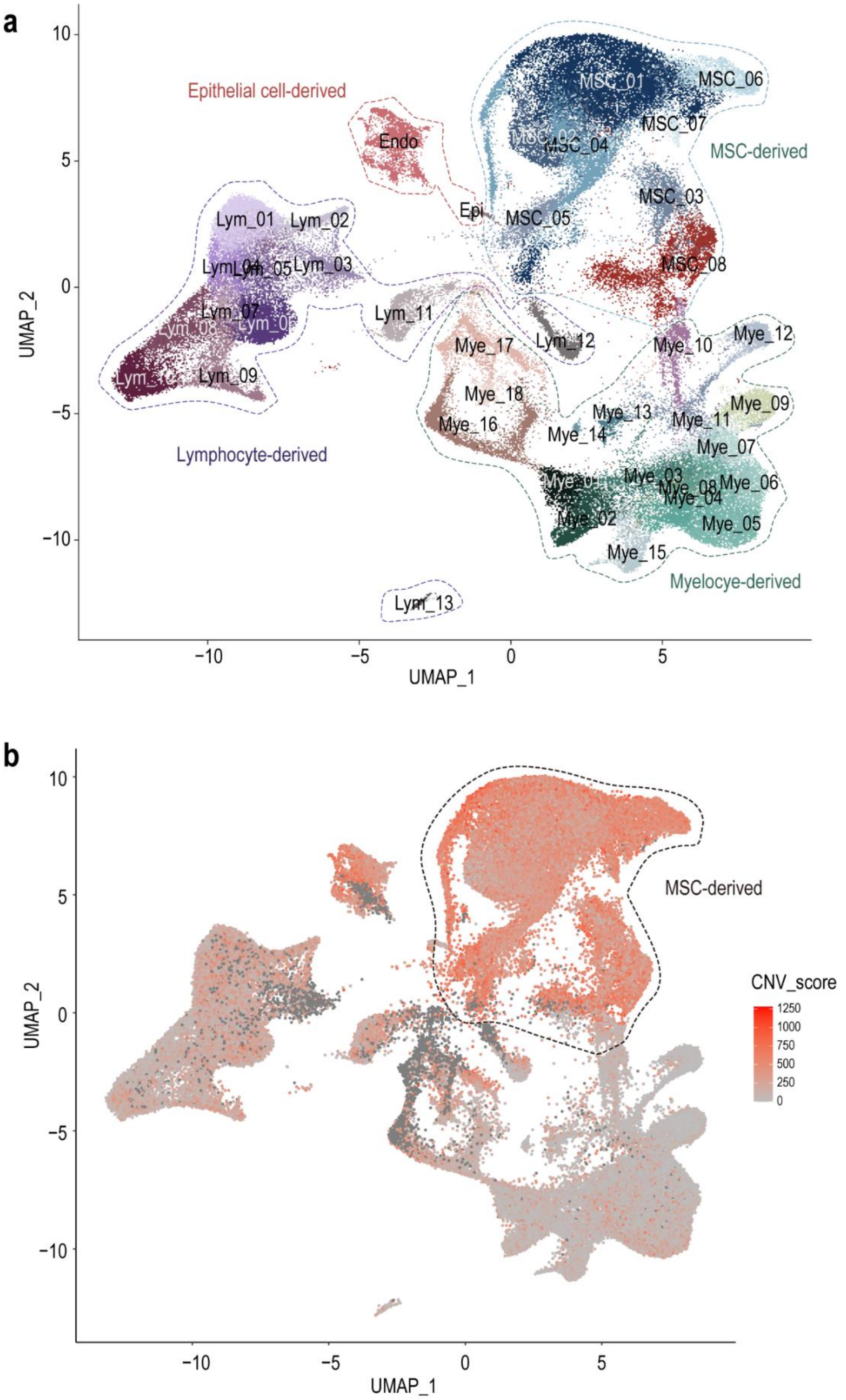

Supplementary Figure 1: UMAP dimensionality reduction plot of the single-cell dataset, related to Figure 3.

(a) High-resolution single-cell atlas of the osteosarcoma tumor microenvironment.

(b) Copy number variation (CNV score) of the osteosarcoma tumor microenvironment.

Figure S2

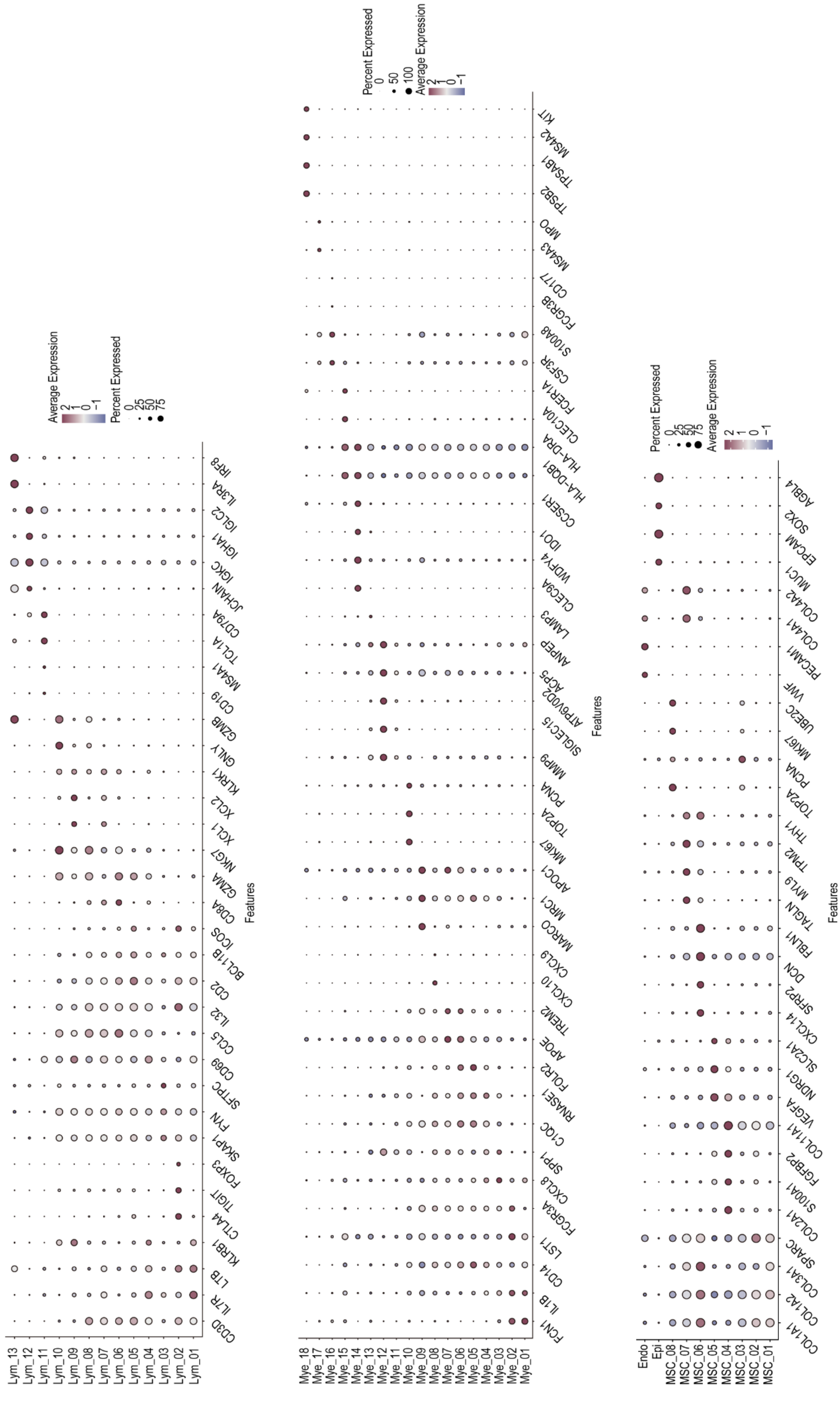

Supplementary Figure 2: Cell annotation characteristics of each cell subgroups in high-resolution single-cell atlas, related to Figure 3.

Figure S3

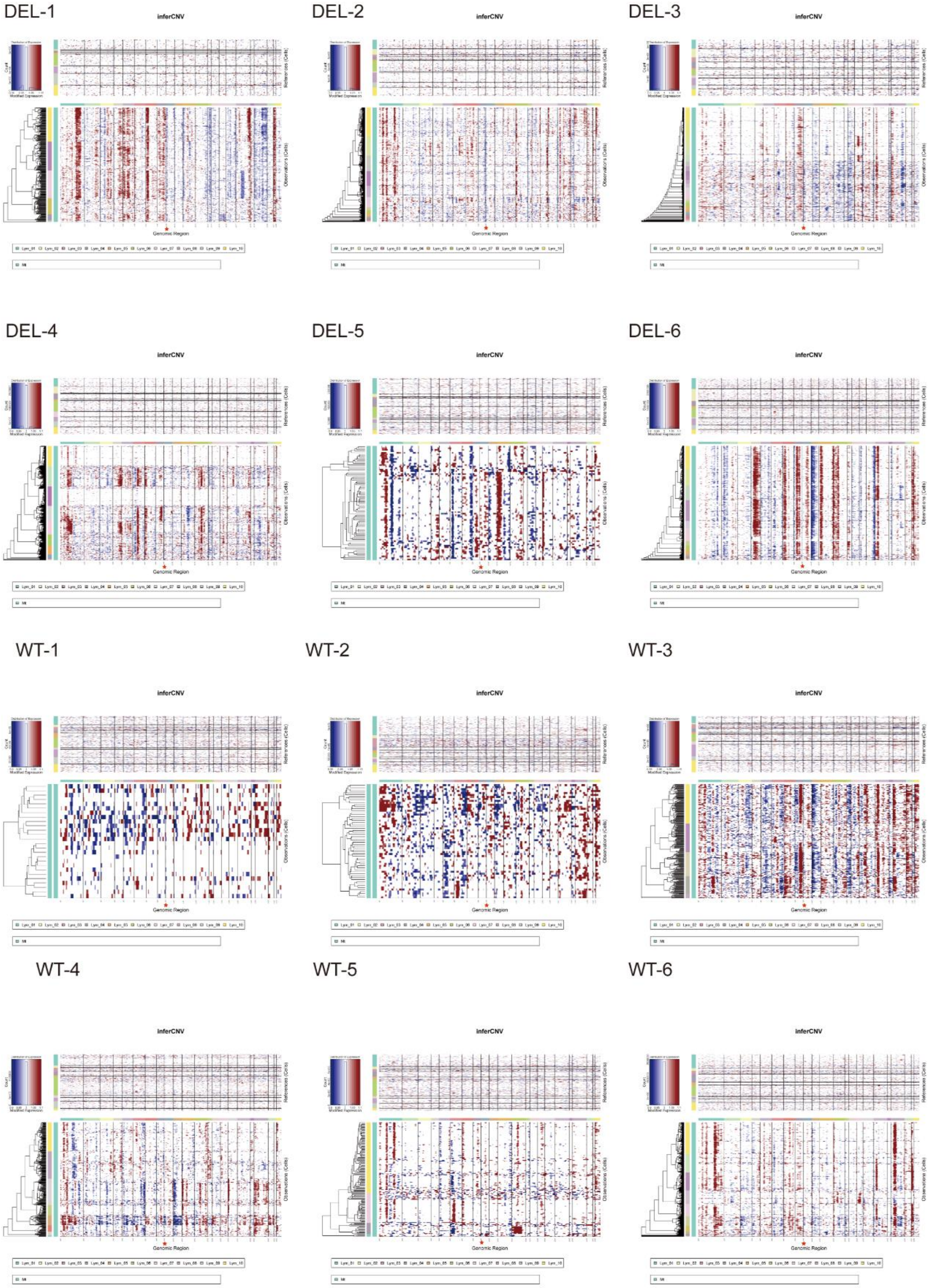

Supplementary Figure 3: Copy number variation of each case by inferCNV. Chr 9p21.3 in each sample was marked with a red asterisk, using MSC_08 cell cluster, compared with Lym_01 to Lym_10, related to Figure 3.

Figure S4

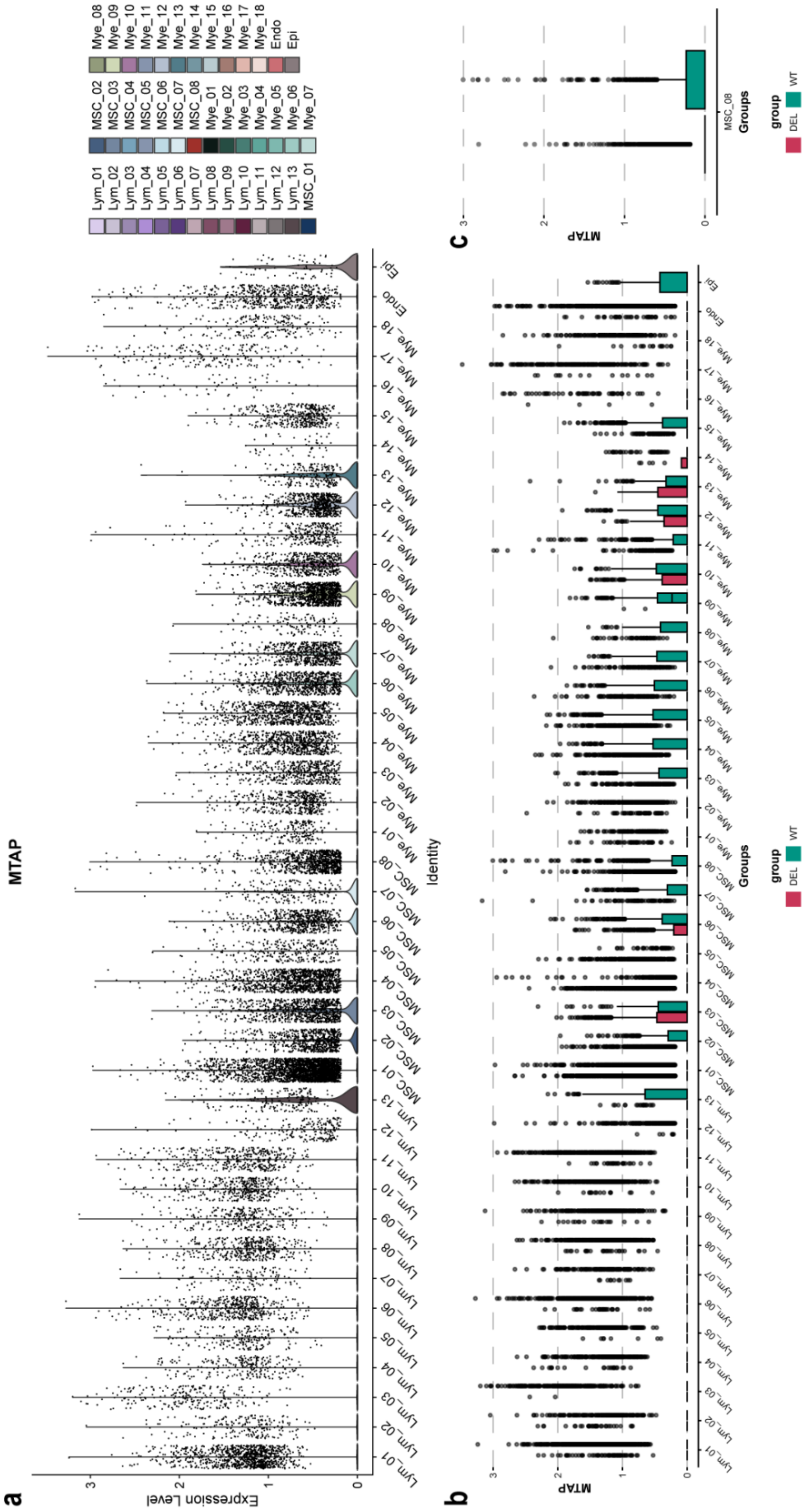

Supplementary Figure 4: MTAP in the osteosarcoma microenvironment, related to Figure 3.

(a) MTAP expression in each cell clusters.

(b) MTAP expression in each cell cluster, separated by group (DEL and WT).

(c) MTAP expression in MSC_08 subtype, separated by group (DEL and WT).

Figure S5

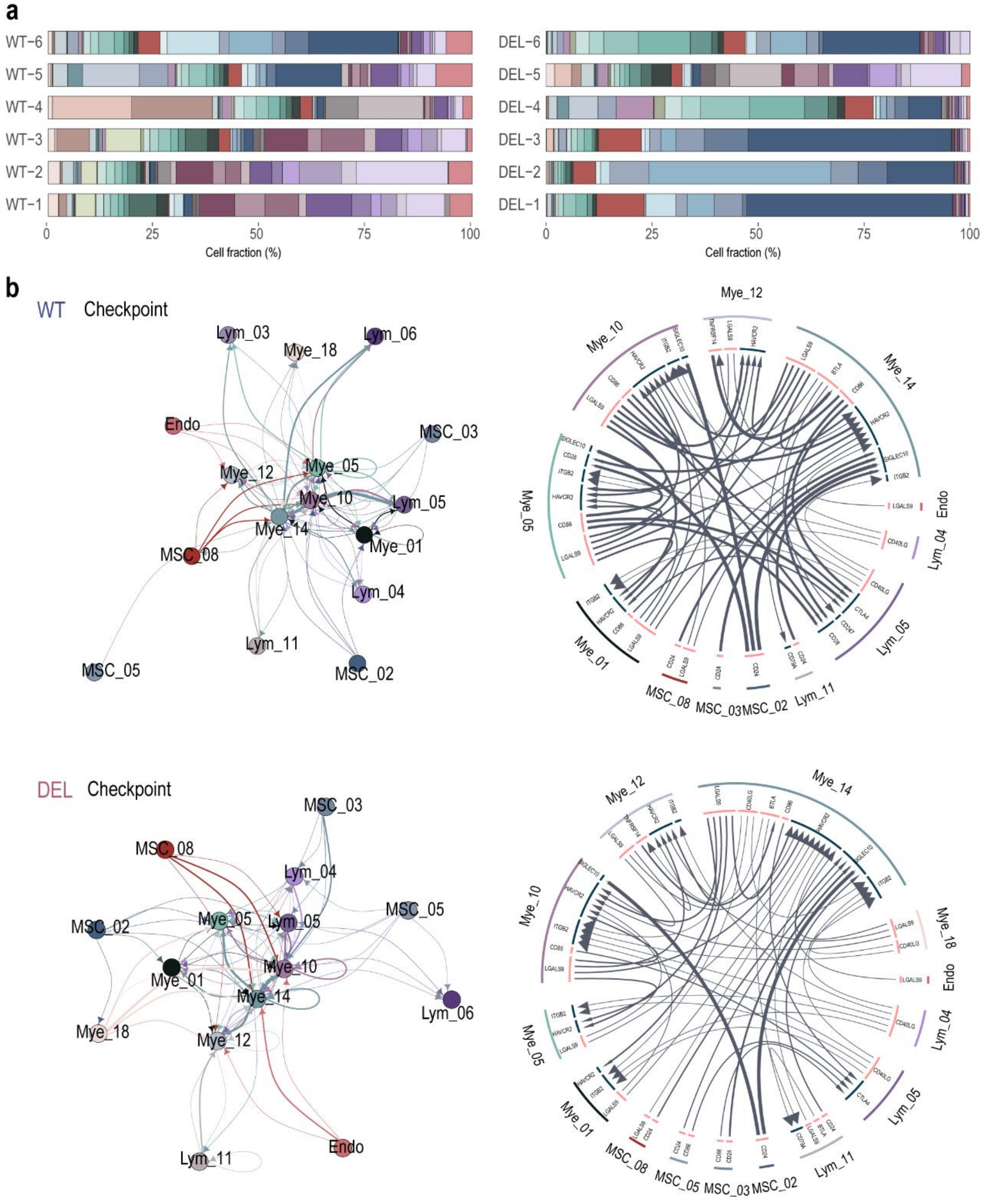

Supplementary Figure 5: Cell distribution and cell communication in the single-cell dataset, related to Figure 3.

(a) Cell components of each case in wild-type (WT) and MTAP-deleted (DEL) osteosarcoma.

(b) Cell-cell communication mediated by receptor-ligand pairs relevant to immune checkpoint therapy by iTalk.

Figure S6

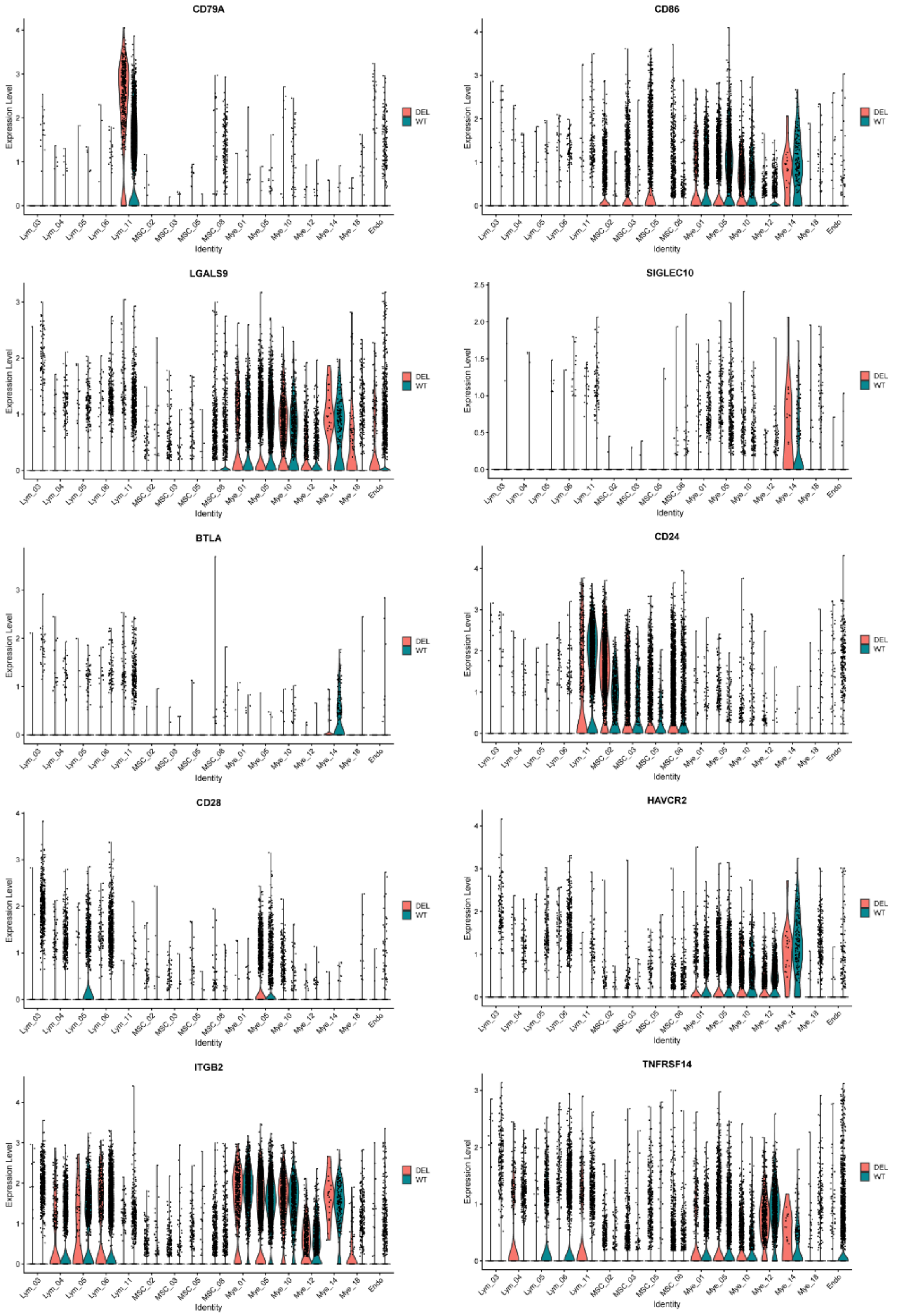

Continuing from Figure S6

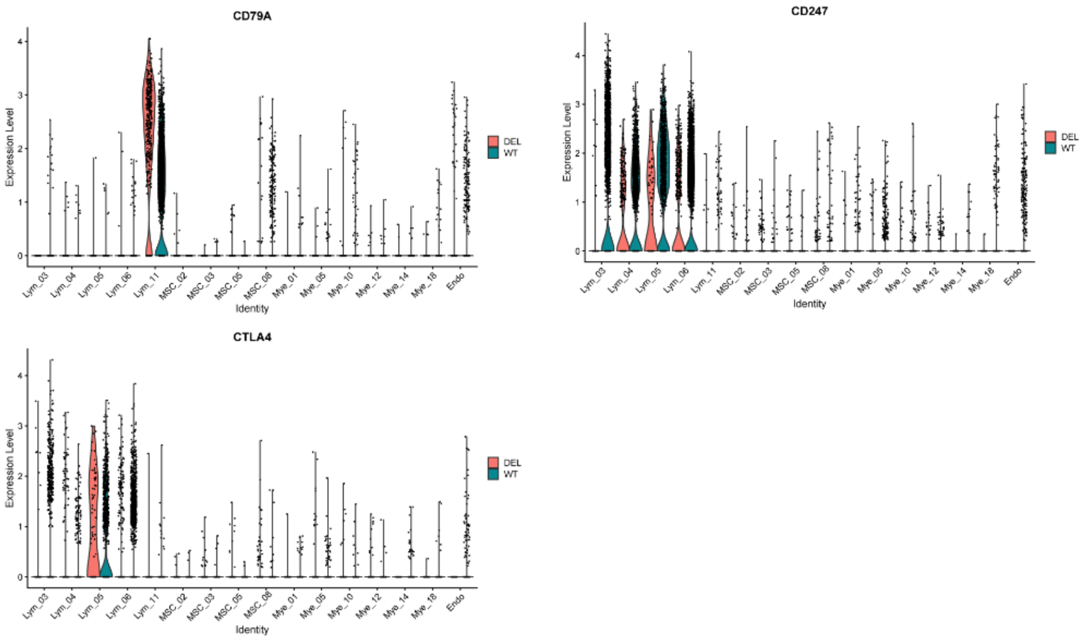

Supplementary Figure 6: Each receptor and ligand in Figure S5b separated by group (DEL and WT), related to Figure 3.

Figure S7

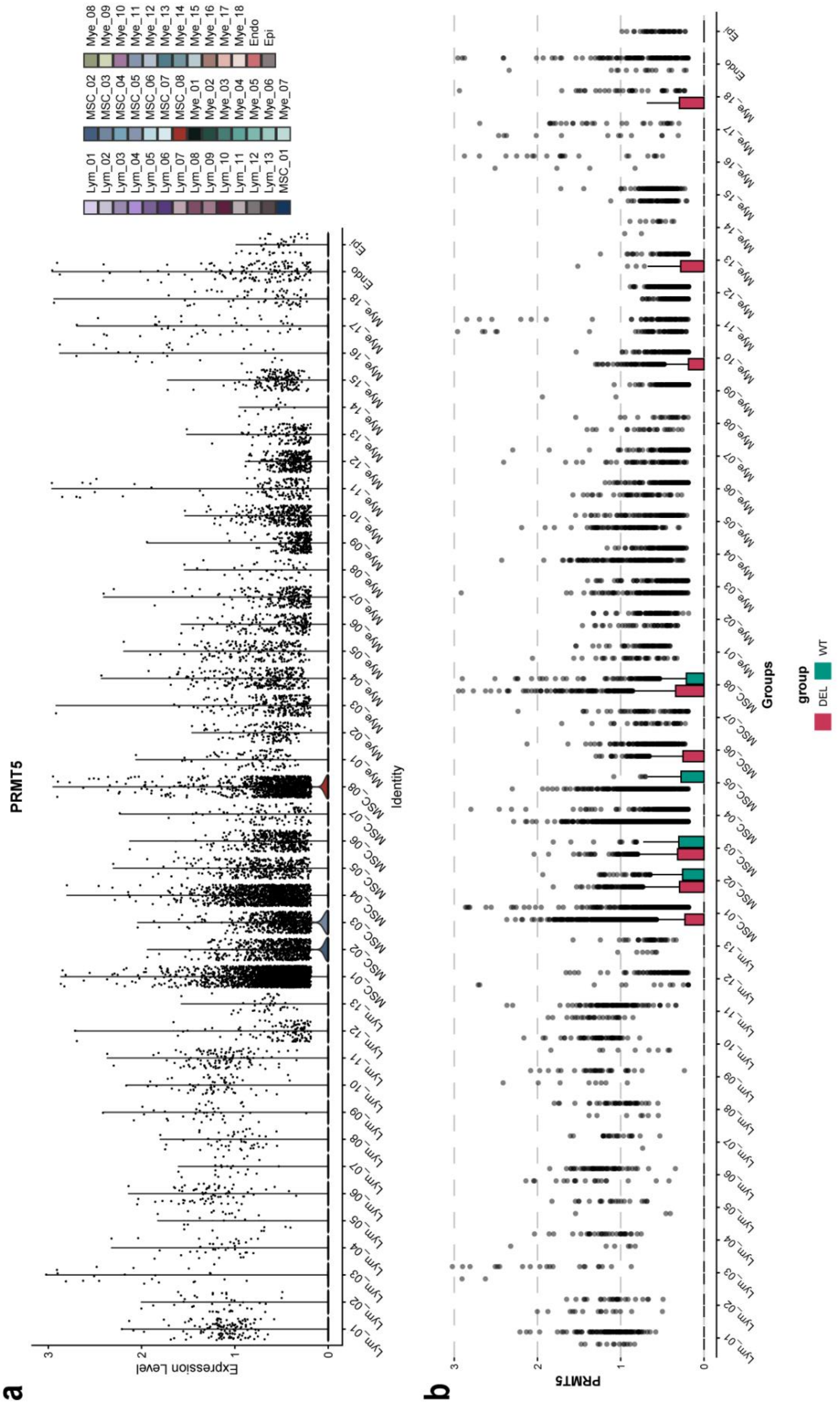

Supplementary Figure 7: PRMT5 in the osteosarcoma microenvironment, related to Figure 3.

(a) PRMT5 expression in each cell clusters.

(b) PRMT5 expression in each cell cluster, separated by group (DEL and WT).

Figure S8

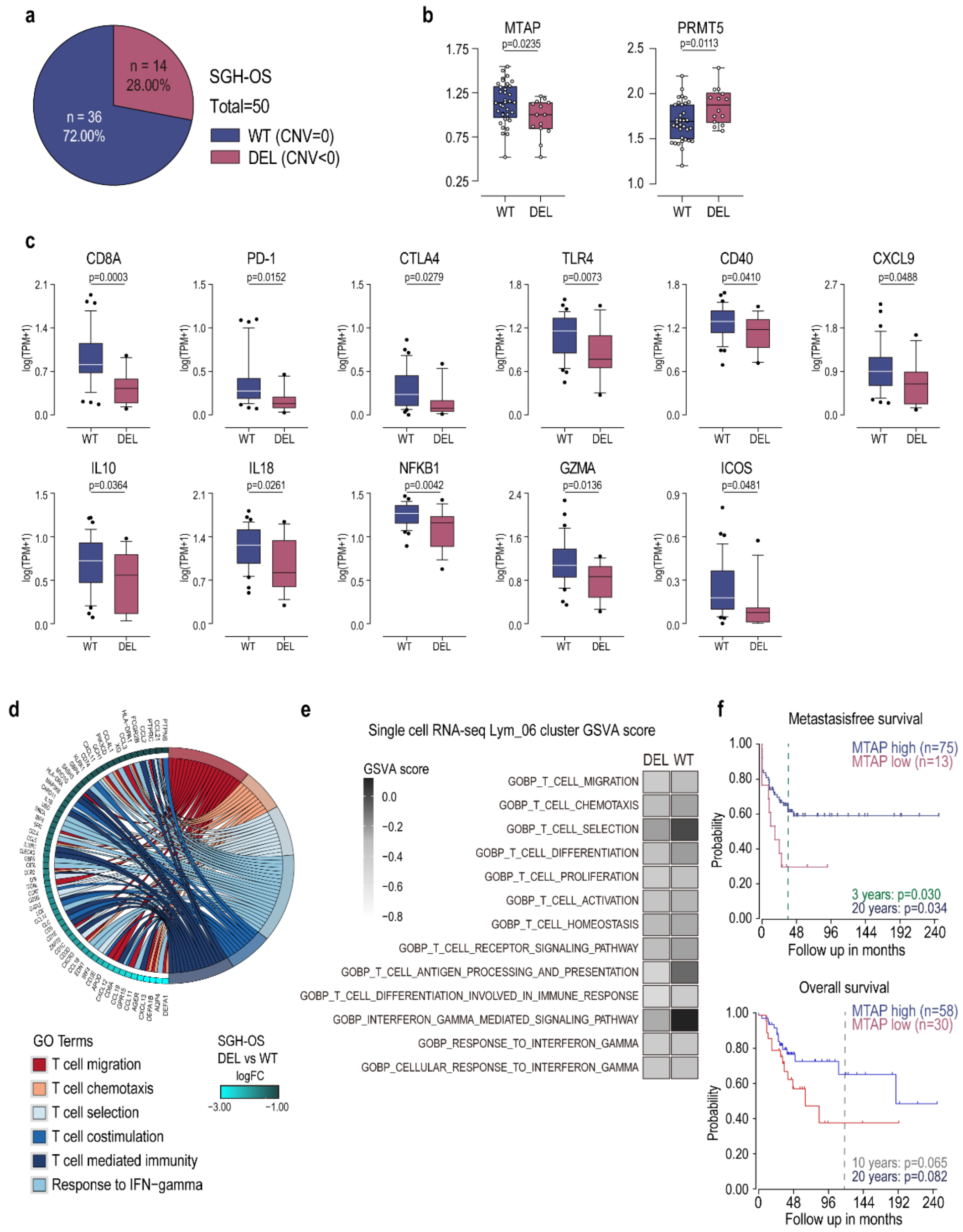

Supplementary Figure 8: MTAP and related genes in SGH-OS cohort, related to Figure 4.

(a) Genomic features of MTAP status in the SGH-OS cohort by methylation array.

(b and c) Transcriptomic features of MTAP and immune-related genes matched with genomic features in SGH-OS. Statistical analyses were performed using two-tailed Student's t-tests.

(d) Signaling pathway features of MTAP-deleted osteosarcoma (DEL) compared with wild-type (WT), using genes logFC < 0.

(e) GSVA scores of T cell function-related pathways derived from scRNA-seq data. DEL, MTAP-deleted osteosarcoma; WT, MTAP wild-type osteosarcoma.

(f) Survival curve of metastasisfree survival and overall survival related with MTAP expression. Statistical significance was calculated using the Log-Rank test.

Figure S9

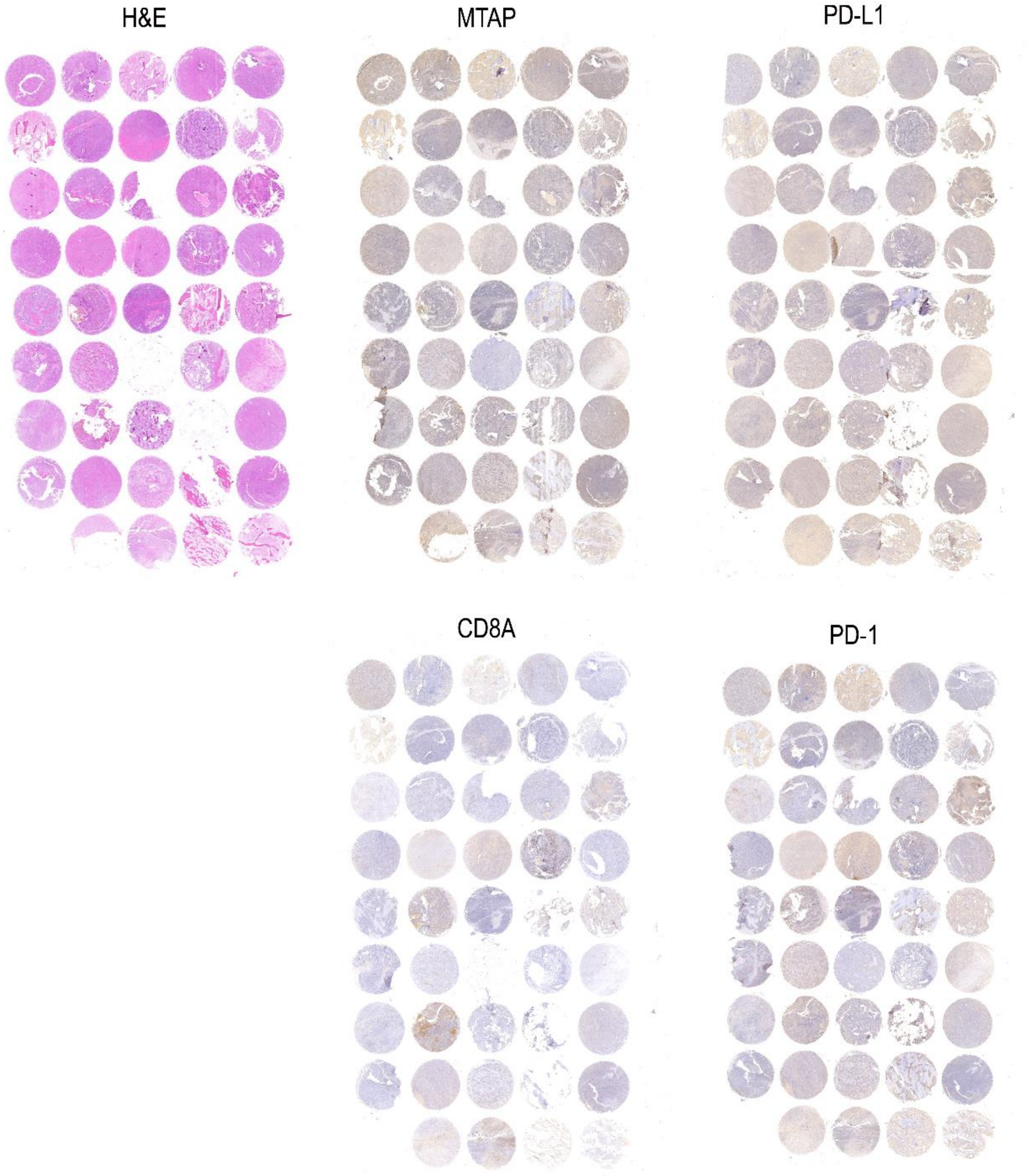

E	D	C	B	A	
DEL	WT	WT	DEL	WT	1
WT	DEL	WT	DEL	WT	2
WT	WT	WT	WT	DEL	3
WT	WT	WT	Unknown	DEL	4
DEL	DEL	DEL	WT	WT	5
WT	DEL	Unknown	DEL	DEL	6
WT	WT	DEL	DEL	WT	7
Unknown	DEL	WT	DEL	WT	8
	WT	Unknown	Unknown	DEL	9

Supplementary Figure 9: Information of tissue microarray. WT: wild-type, DEL: MTAP-deleted, related to Figure 4.

Figure S10

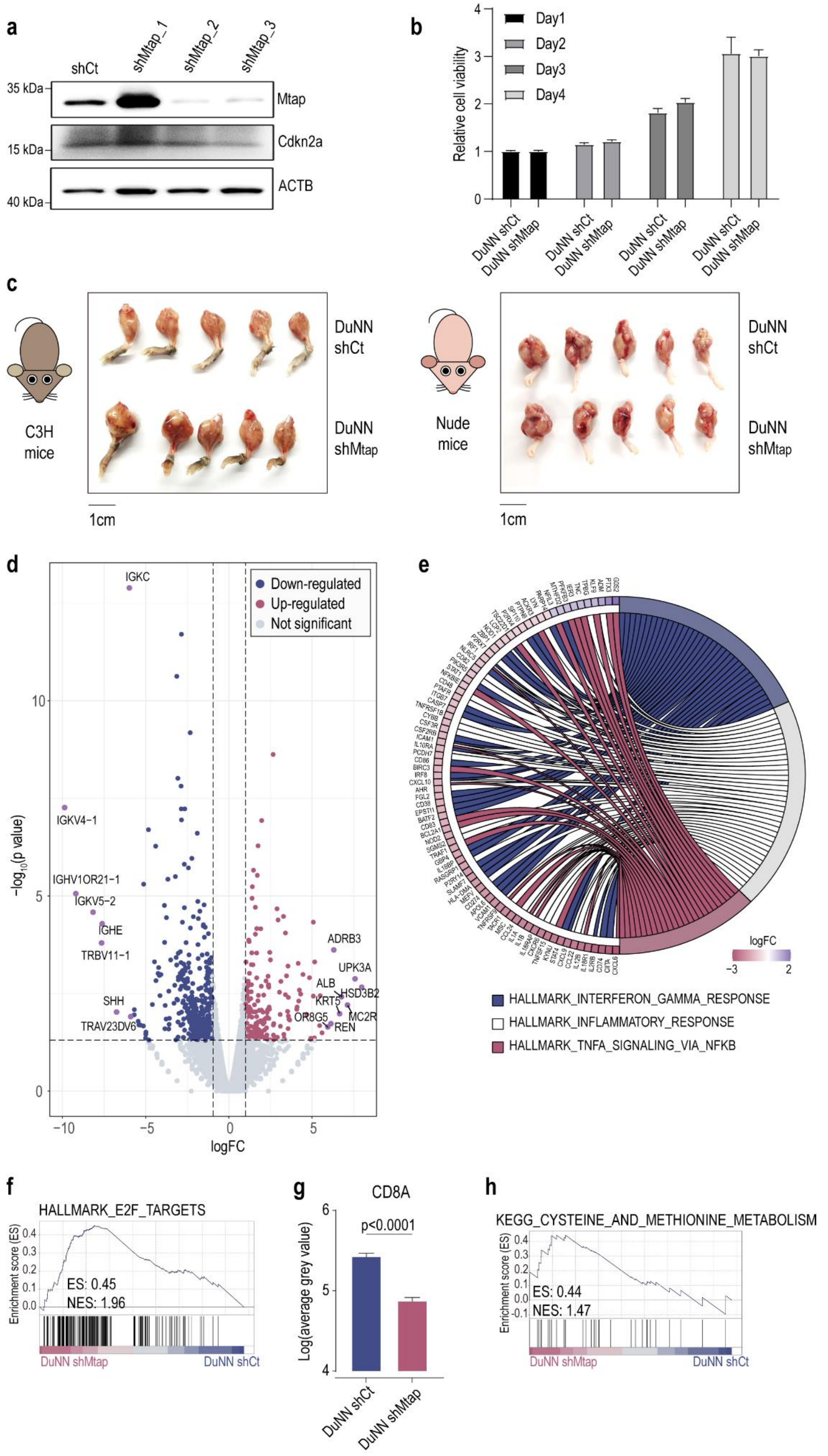
a
shCt
shMtap_1
shMtap_2
shMtap_3
35 kDa
15 kDa
40 kDa
Mtap
Cdkn2a
ACTB
b
Relative cell viability
Day1
Day2
Day3
Day4
DuNN shCt
DuNN shMtap
c
C3H mice
Nude mice
DuNN shCt
DuNN shMtap
1cm
d
Down-regulated
Up-regulated
Not significant
IGKC
IGKV4−1
IGHV1OR21−1
IGKV5−2
IGHE
TRBV11−1
SHH
TRAV23DV6
ADRB3
UPK3A
ALB
HSD3B2
KRT5
OR8G5
MC2R
REN
−log10(p value)
logFC
e
logFC
HALLMARK_INTERFERON_GAMMA_RESPONSE
HALLMARK_INFLAMMATORY_RESPONSE
HALLMARK_TNFA_SIGNALING_VIA_NFKB
f
HALLMARK_E2F_TARGETS
Enrichment score (ES)
ES: 0.45
NES: 1.96
DuNN shMtap
DuNN shCt
g
CD8A
p<0.0001
Log(average grey value)
DuNN shCt
DuNN shMtap
h
KEGG_CYSTEINE_AND_METHIONINE_METABOLISM
Enrichment score (ES)
ES: 0.44
NES: 1.47
DuNN shMtap
DuNN shCt

Supplementary Figure 10: In vivo experiment validating the role of MTAP loss in immune suppression in osteosarcoma, related to Figure 5.

(a) Protein expression of Mtap and Cdkn2a in DuNN shMtap and DuNN shCt models, using shMtap_2 for further investigations in our study.

(b) Cell viability of DuNN shMtap and DuNN shCt *in vitro*. Technical replicates, n = 3.

(c) Gross specimens of experiments in Figure 5a and 5b.

(d and e) Transcriptomic analysis related to Figure 5c.

(f) Transcriptomic pathway analysis of tumor tissues from DuNN shMTAP and DuNN shCt in C3H mice, using GSEA analysis on Hallmark gene set (E2F_TARGETS).

(g) Quantitation of immunohistochemistry, related to Figure 5d. Statistical analyses were performed using two-tailed Student's t-tests. Technical replicates, n = 3.

(h) Transcriptomic pathway analysis of tumor tissues from DuNN shMTAP and DuNN shCt in C3H mice, using GSEA analysis on KEGG gene set (CYSTENE _ AND _ METHIONINE _ METABOLISM).

ES refers to enrichment score, and NES refers to normalized enrichment score.

Figure S11

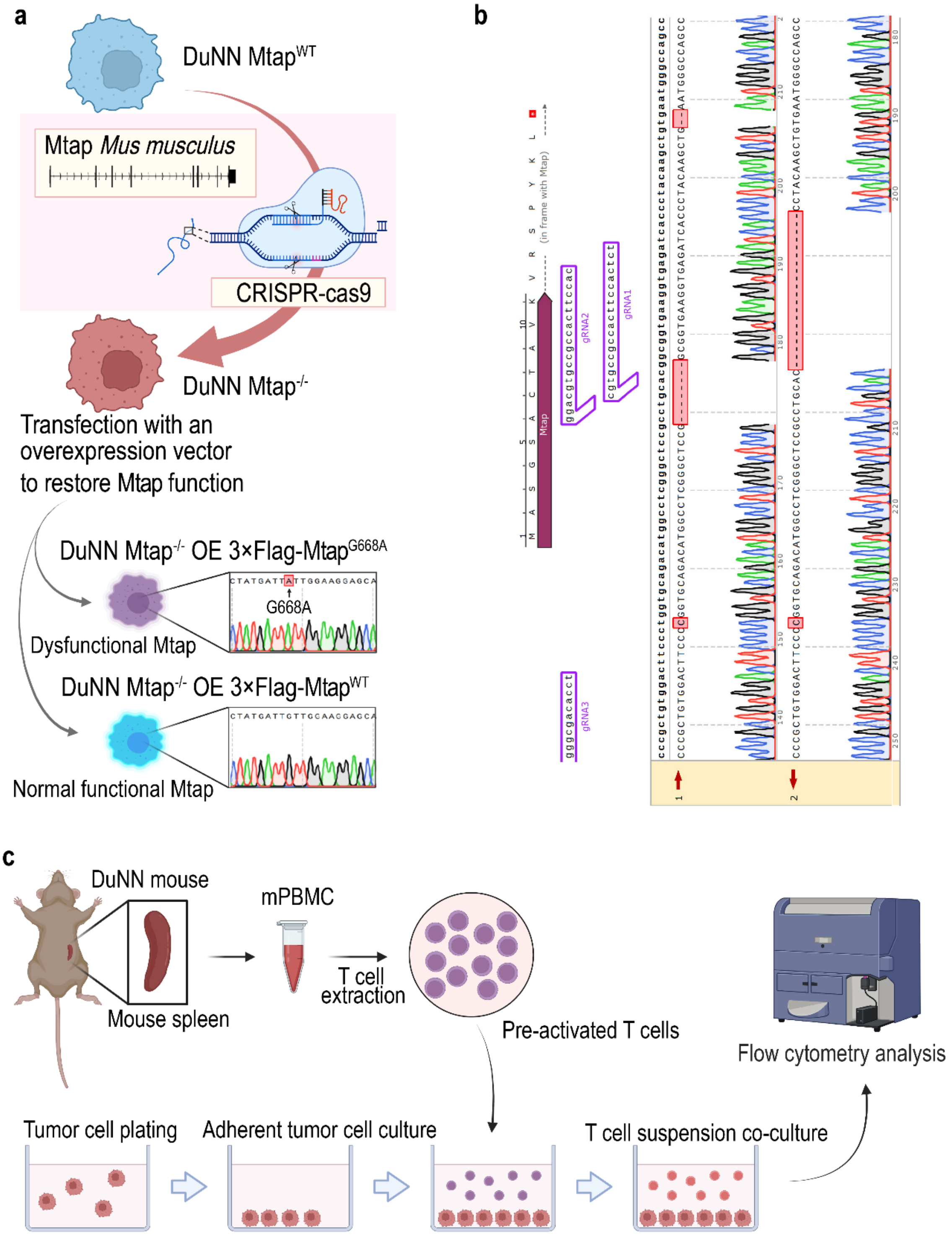

Supplementary Figure 11: Gene editing of DuNN Mtap$^{-/-}$ and the co-culture process, related to Figure 5.

(a) Schematic diagram of Mtap knockout in DuNN and rescue experiments. Genomic PCR confirmed Mtap^{G668A} mutation, where G at position 668 was mutated to A.

(b) Genomic PCR of Mtap$^{-/-}$ cas9 gRNA.

(c) Schematic diagram showing the co-culture of DuNN tumor cells and T cells.

Figure S12

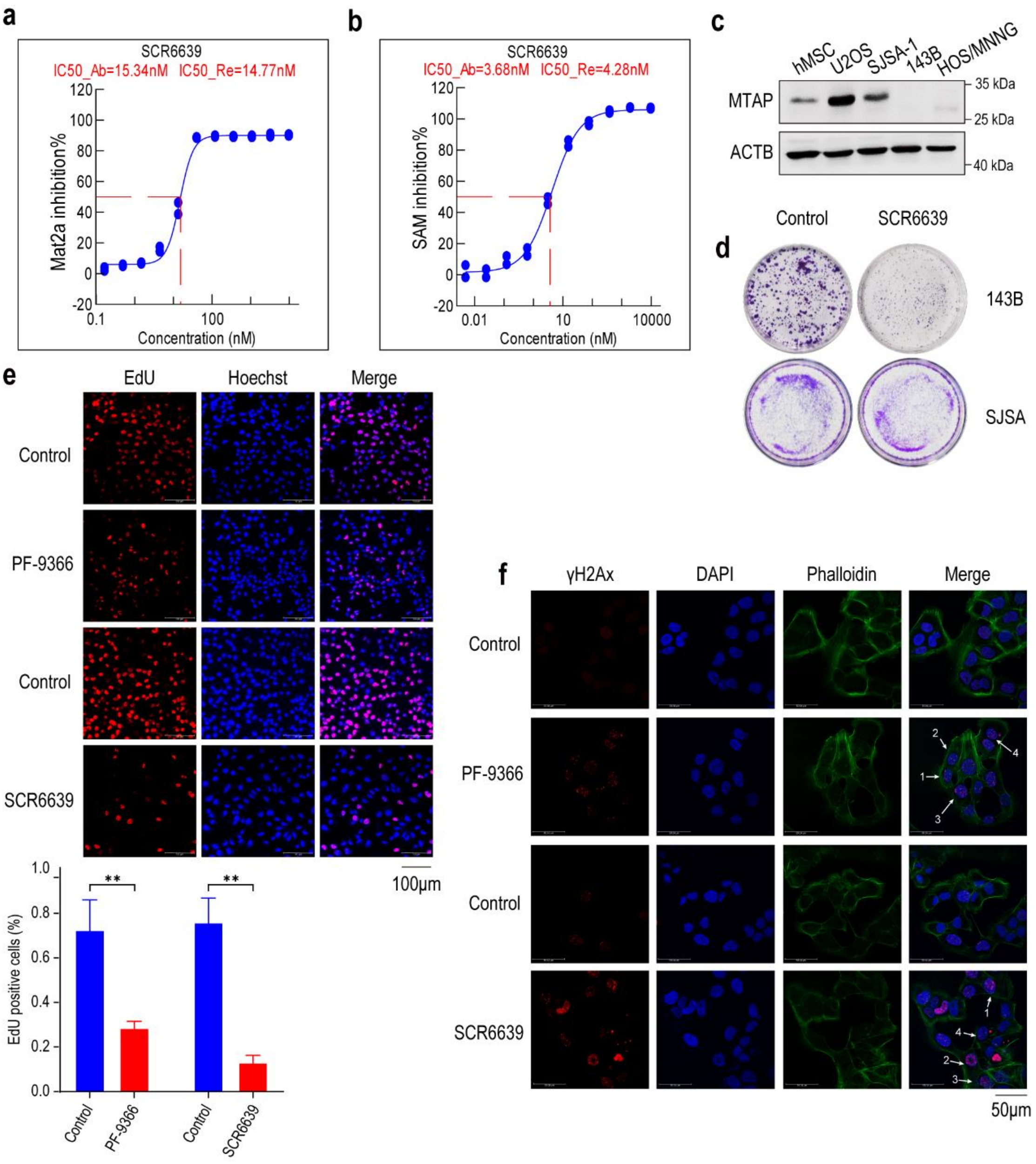

Supplementary Figure 12: ***In vitro*** **functional assay of MAT2A inhibitor SCR6639, related to Figure 6.**

(a) Assessment of SCR6639's ability to inhibit Mat2a enzymatic activity**.** Technical replicates, n = 2.

(b) Assessment of SCR6639's ability to inhibit SAM synthesis. Technical replicates, n = 2.

(c) The protein expression of MTAP in human MSC and osteosarcoma cell lines by western blotting

(d) Inhibition of MAT2A selectively induces cytotoxicity in MTAP-deleted OS cell lines.

(e) MAT2A inhibitor suppresses the *in vitro* proliferation of MTAP-deleted OS cells. Statistical analyses were performed using two-tailed Student's t-tests. Technical replicates, n = 3. **: p value < 0.01.

(f) The MAT2A inhibitor can induce DNA damage in MTAP-deleted OS cells.

Figure S13

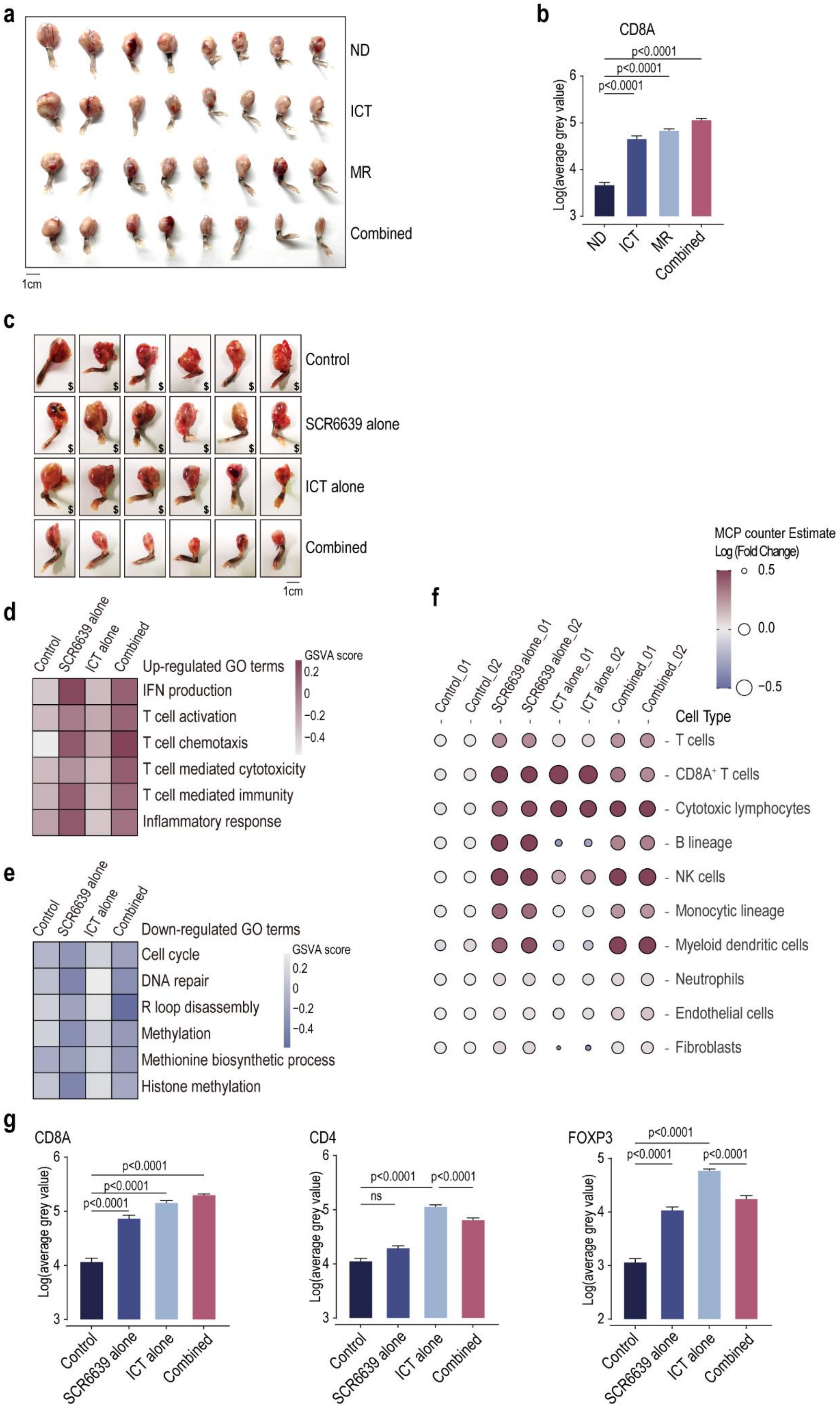
a
ND
ICT
MR
Combined
1cm
b
CD8A
p<0.0001
p<0.0001
p<0.0001
Log(average grey value)
ND
ICT
MR
Combined
c
Control
SCR6639 alone
ICT alone
Combined
1cm
d
Control
SCR6639 alone
ICT alone
Combined
Up-regulated GO terms
IFN production
T cell activation
T cell chemotaxis
T cell mediated cytotoxicity
T cell mediated immunity
Inflammatory response
GSVA score
e
Down-regulated GO terms
Cell cycle
DNA repair
R loop disassembly
Methylation
Methionine biosynthetic process
Histone methylation
GSVA score
f
MCP counter Estimate
Log (Fold Change)
Control_01
Control_02
SCR6639 alone_01
SCR6639 alone_02
ICT alone_01
ICT alone_02
Combined_01
Combined_02
Cell Type
T cells
CD8A⁺ T cells
Cytotoxic lymphocytes
B lineage
NK cells
Monocytic lineage
Myeloid dendritic cells
Neutrophils
Endothelial cells
Fibroblasts
g
CD8A
CD4
FOXP3
p<0.0001
ns
Log(average grey value)
Control
SCR6639 alone
ICT alone
Combined

Supplementary Figure 13: In vivo experiment of methionine intervention combined with ICT, related to Figure 6.

(a) Gross specimens of experiments in Figure 6a.

(b) Quantitation of immunohistochemistry, related to Figure 6e. Statistical analyses were performed using two-tailed Student's t-tests. Technical replicates, n = 3.

(c) Gross specimens of experiments in Figure 6f.

(d and e) Transcriptomic pathway analysis of tumor tissues from DuNN shMTAP in C3H mice treated by MAT2A inhibition combined with immune checkpoint therapy, using GSVA analysis on GO gene sets.

(f) Estimating cell fraction of immune cells and stromal cells in MAT2A inhibition combined with immune checkpoint therapy, using MCP counter.

(g) Quantitation of immunohistochemistry, related to Figure 6i. Statistical analyses were performed using two-tailed Student's t-tests. Technical replicates, n = 3. ns: not statistically significant.

Figure S14

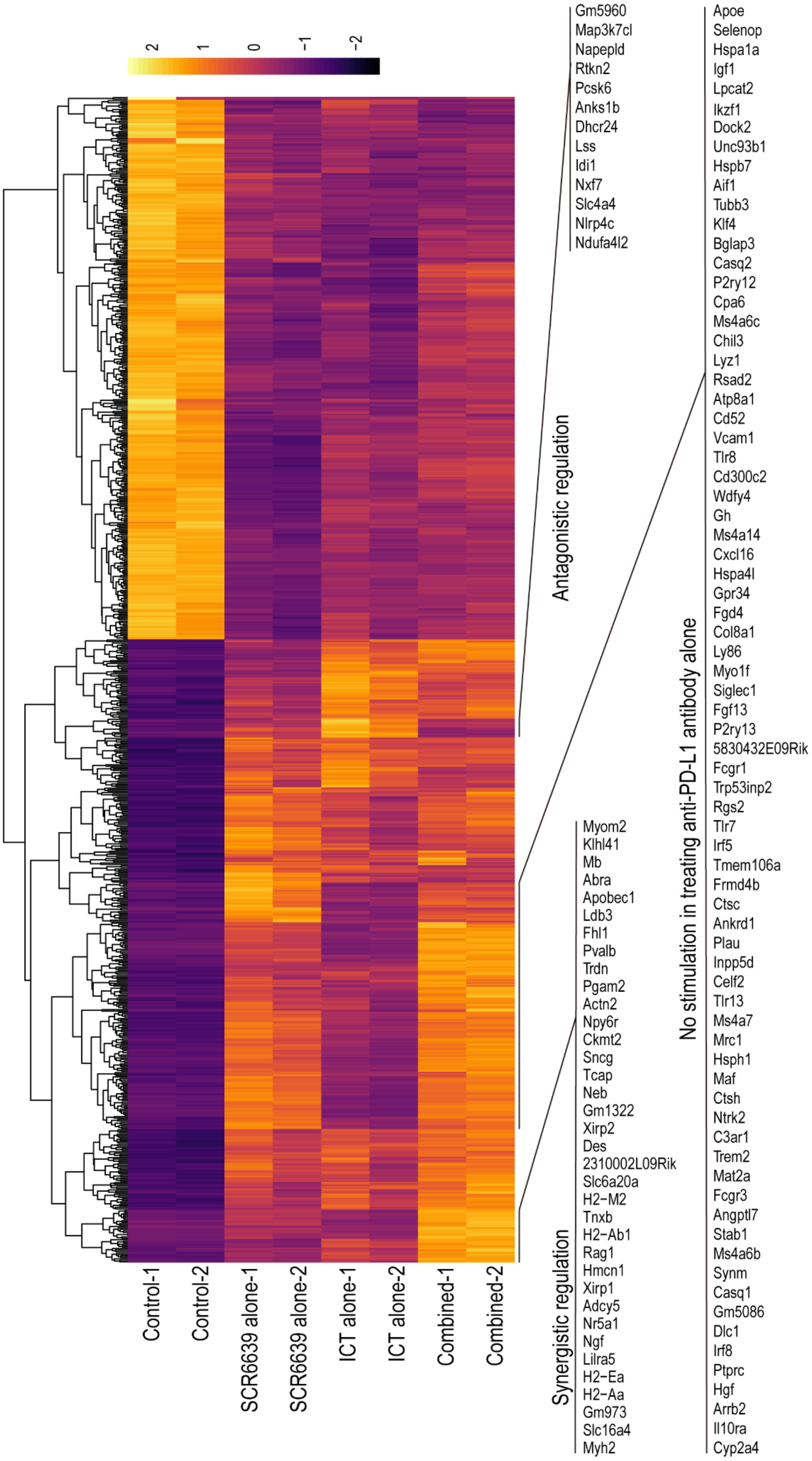

Supplementary Figure 14: The differential gene expression analysis from bulk RNA-seq data, treated by MAT2A inhibition combined with immune checkpoint therapy, related to Figure 6.

Figure S15

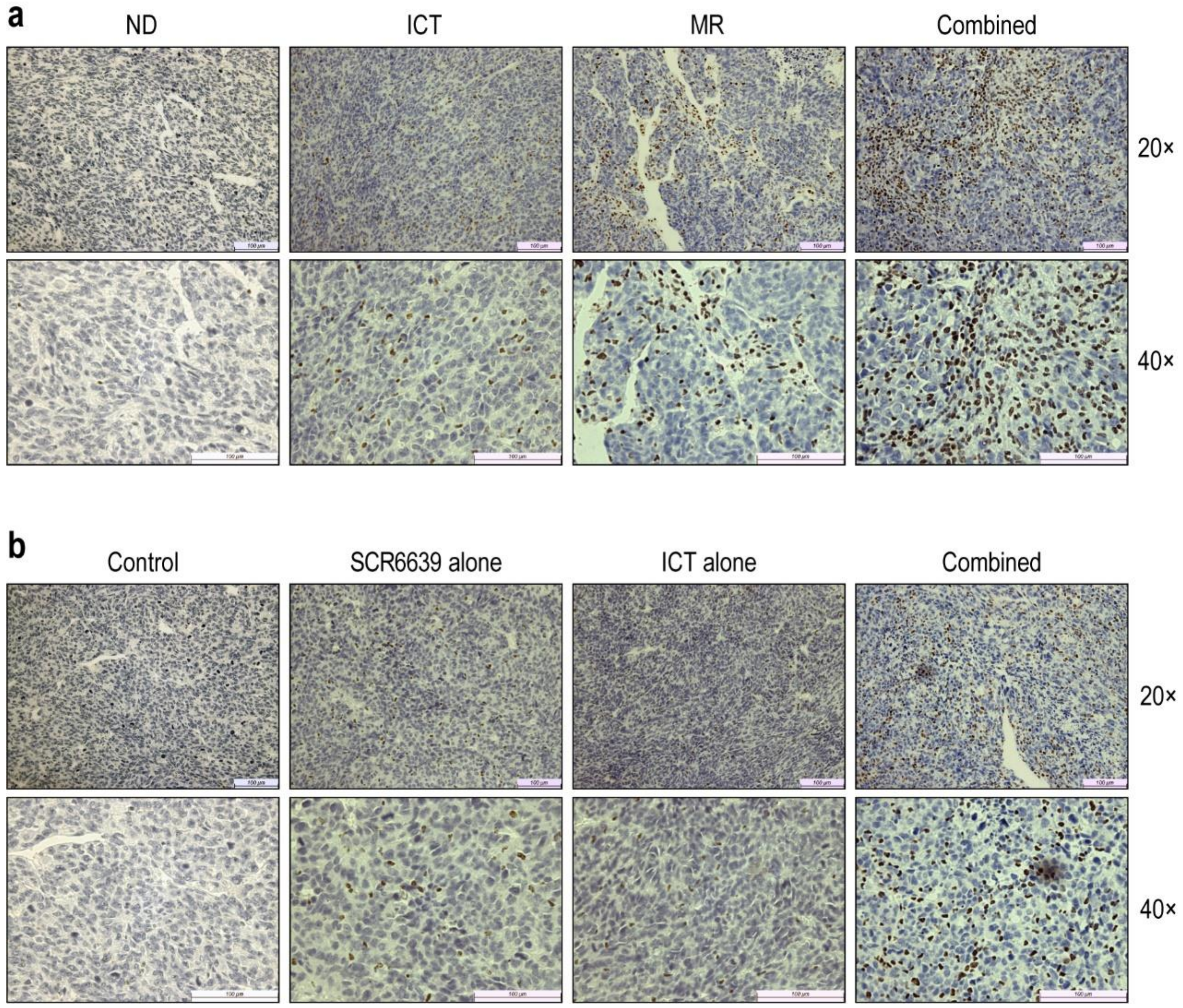

Supplementary Figure 15: In vivo experiment IHC of IKZF1, related to Figure 6.

(a) IHC staining of IKZF1 expression in tumor tissues following treatment with a methionine-restricted diet combined with ICT. Scale bar = 100 μm.

(b) IHC staining of IKZF1 expression in tumor tissues following treatment with SCR6639, a MAT2A inhibitor, combined with ICT. Scale bar = 100 μm.

Figure S16

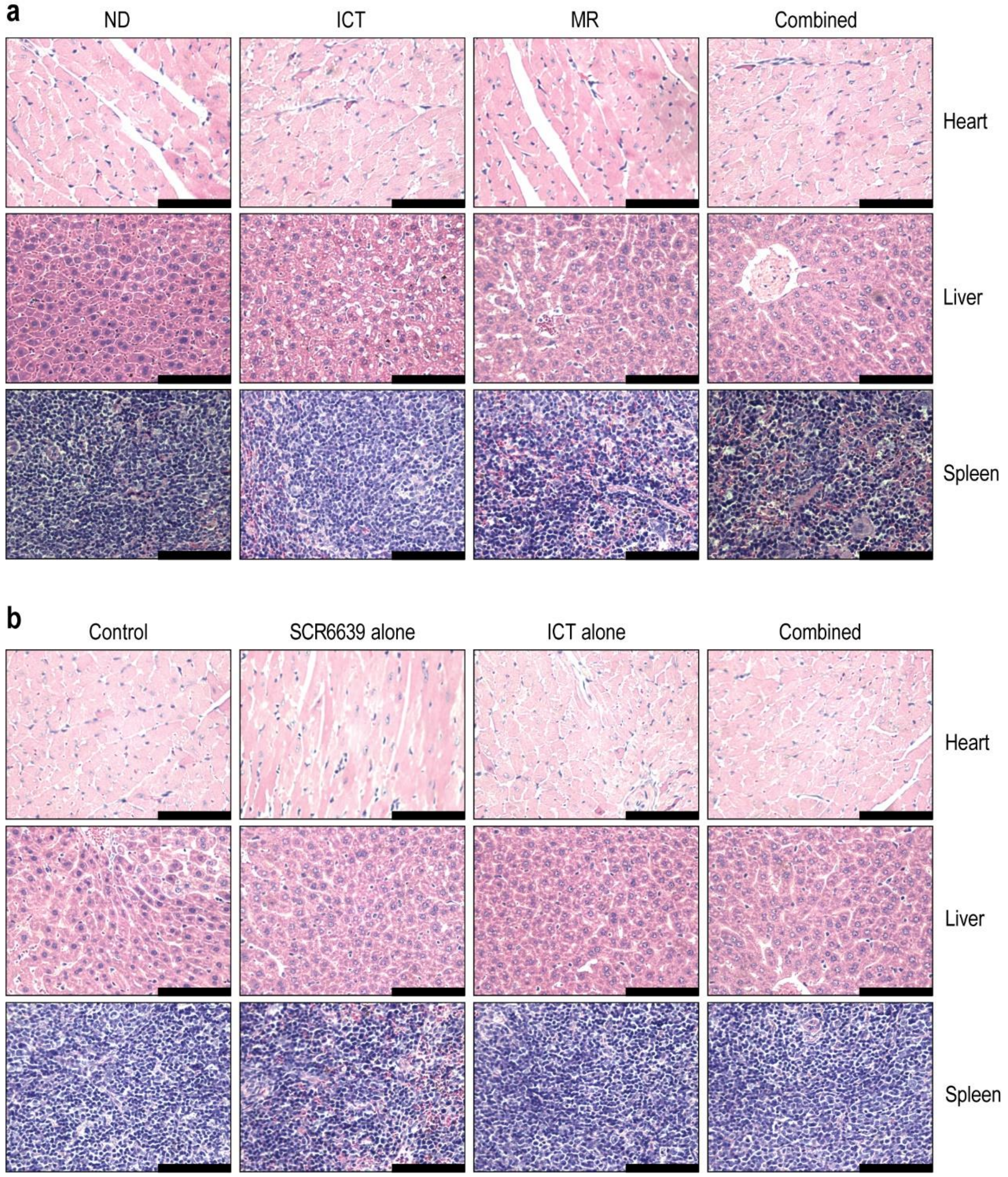

Supplementary Figure 16: Histopathological staining of the heart, liver, and spleen from experimental animals in the in vivo experiment, related to Figure 6.

(a) H&E staining of histopathological features in heart, liver and spleen following treatment with a methionine-restricted diet combined with ICT. The combination treatment showed no significant toxicity. Scale bar = 100 μm.

(b) H&E staining of histopathological features in heart, liver and spleen following treatment with SCR6639, a MAT2A inhibitor, combined with ICT. The combination treatment showed no significant toxicity. Scale bar = 100 μm.
